# Safety of transcutaneous auricular vagus nerve stimulation (taVNS): a systematic review and meta-analysis

**DOI:** 10.1038/s41598-022-25864-1

**Published:** 2022-12-21

**Authors:** Angela Yun Kim, Anna Marduy, Paulo S. de Melo, Anna Carolyna Gianlorenco, Chi Kyung Kim, Hyuk Choi, Jae-Jun Song, Felipe Fregni

**Affiliations:** 1 Department of Otorhinolaryngology-Head and Neck Surgery, Korea University Medical Center, Seoul, Republic of Korea; 2 Uniao Metropolitana de Ensino e Cultura (UNIME) Salvador, Bahia, Brazil; 3 Neuromodulation Center and Center for Clinical Research Learning, Spaulding Rehabilitation Hospital and Massachusetts General Hospital, Harvard Medical School, 96 13th Street, Charlestown, Boston, MA USA; 4 Department of Medicine, Escola Bahiana de Medicina e Saude Publica, Salvador, Bahia Brazil; 5 Department of Physical Therapy, Federal University of Sao Carlos, Sao Carlos, Brazil; 6 Department of Neurology, Korea University Guro Hospital, Seoul, Republic of Korea; 7 Department of Medical Sciences, Graduate School of Medicine, Korea University, Seoul, Republic of Korea; 8 Neurive Co., Ltd., Gimhae, Republic of Korea

**Keywords:** Outcomes research, Neuroscience, Medical research, Neurology

## Abstract

Transcutaneous auricular vagus nerve stimulation (taVNS) has been investigated as a novel neuromodulation tool. Although taVNS is generally considered safe with only mild and transient adverse effects (AEs), those specifically caused by taVNS have not yet been investigated. This systematic review and meta-analysis on taVNS aimed to (1) systematically analyze study characteristics and AE assessment, (2) characterize and analyze possible AEs and their incidence, (3) search for predictable risk factors, (4) analyze the severity of AE, and (5) suggest an evidence-based taVNS adverse events questionnaire for safety monitoring. The articles searched were published through April 7, 2022, in Medline, Embase, Web of Science, Cochrane, and Lilacs databases. In general, we evaluated 177 studies that assessed 6322 subjects. From these, 55.37% of studies did not mention the presence or absence of any AEs; only 24.86% of the studies described that at least one adverse event occurred. In the 35 studies reporting the number of subjects with at least one adverse event, a meta-analytic approach to calculate the risk differences of developing an adverse event between active taVNS and controls was used. The meta-analytic overall adverse events incidence rate was calculated for the total number of adverse events reported on a 100,000 person-minutes-days scale. There were no differences in risk of developing an adverse event between active taVNS and controls. The incidence of AE, in general, was 12.84/100,000 person-minutes-days of stimulation, and the most frequently reported were ear pain, headache, and tingling. Almost half of the studies did not report the presence or absence of any AEs. We attribute this to the absence of AE in those studies. There was no causal relationship between taVNS and severe adverse events. This is the first systematic review and meta-analysis of transcutaneous auricular stimulation safety. Overall, taVNS is a safe and feasible option for clinical intervention.

## Introduction

The vagus nerve is a mixed nerve composed of 20% efferent fibers and 80% afferent fibers^1^. It plays a major role in maintaining autonomic tone throughout in the brain, the thorax, and the abdomen. The vagus nerve stimulation (VNS), first approved by the US Food and Drug Association (FDA) in 1997 in the form of a cervical implantable device, is currently approved for drug-resistant epilepsy, depression, and morbid obesity^2,3^. Further studies have shown promising results that VNS can treat disorders such as anxiety disorder, Alzheimer’s disease, cluster headache, heart failure, sepsis, lung injury, rheumatoid arthritis, inflammatory bowel disease, obesity, and diabetes^4–6^.

However, this classic approach requires an invasive procedure that involves cervical dissection and foreign body implantation. Due to the potential risks of surgical complications, hospitalization cost, and accessibility, the application of the intervention is limited despite its demonstrated benefits. Thin-myelinated afferent fibers of the cervical vagus nerve are targets for clinically effective VNS^6^. However, it is impossible to selectively stimulate certain fibers through conventional methods, and efferent branches are unintentionally stimulated together. Efferent branches send parasympathetic cholinergic signals to target organs, including the lungs, digestive tract, and heart^3^. The most critical reports include cardiac events, such as bradycardia and asystole, occurring in approximately 1 per 1000 cases as a result of direct stimulation of the cardiac branches of the vagus nerve^7^. Injury to the efferent branches may also cause hoarseness, dyspnea, and dysphagia related to left vocal cord paralysis^8^. Therefore, there has been an increasing demand for a non-invasive alternative method to avoid side effects but achieve similar effects^9^.

Transcutaneous VNS can be approached using two methods, stimulation of either the cervical bundle or the auricular branch^10,11^. Because there is no need for surgical procedures, clinicians in many fields suggest that transcutaneous VNS can be applied to a wider range of patients and is even tolerable for patients with disorders that are not life-threatening. Transcutaneous auricular vagus nerve stimulation (taVNS) is a newer delivery system that uses an electrical transcutaneous stimulation device placed at the concha or tragus of the ear. fMRI studies have shown that when these sites are stimulated, the ipsilateral nucleus of tractus solitarius (NTS) is activated through the vagal projections in the brainstem and forebrain^12^. Opposing to cervical VNS, taVNS uses a physiological pathway to activate the NTS and dorsal motor nucleus, which subsequently sends impulses to the heart surface bilaterally via efferent cervical vagus nerves^13^. Therefore, this technique avoids the possibility of directly and asymmetrically stimulating cardiac motor efferent fibers, which could result in unfavorable cardiac events. The taVNS combines advantageous qualities including non-invasiveness, cost, easiness, and efficacy to make it an appealing intervention for neuropsychiatric illnesses^13^.

Many authors consider taVNS a safer substitute for the previous surgical device^4,14–17^. Thus far, taVNS use has been studied in many conditions such as epilepsy, tinnitus, depression, stroke, Alzheimer’s disease^18^.

Therefore, it is important to assess the safety of taVNS. Although the general impression is that taVNS is a safe technique with only mild and transient adverse effects (AEs), human data on safety and tolerability are largely based on clinical trials with small study populations. A review study in 2018 addressed this issue and was the first to systematically report transcutaneous vagus nerve stimulation treatment harms^11^. The author concluded that transcutaneous vagus nerve stimulation is well tolerated and safe, with only mild side effects such as local skin irritation due to electrode placement, headache, and nasopharyngitis^11^. Overall, 30 serious adverse events were reported (22 participants from 7 studies), but none of them were confirmed to be caused by transcutaneous VNS^11^. However, this study suggests a comprehensive perspective, including transcutaneous vagus nerve stimulation techniques such as that applied to the neck. Adverse effects specifically caused by taVNS have not yet been investigated. In addition, because of the relatively high interest in taVNS, many human studies have been further reported since this publication and require systematic and meta-analytic analysis for possible adverse events. To assess the safety of taVNS under different conditions and study designs, we performed this systematic review and meta-analysis of clinical reports on taVNS.

Another goal of our study was to explore the relationship between the study characteristics and the frequency of adverse events. Because there are no guidelines for taVNS clinical trials, there is a large interstudy heterogeneity in study methodology, device type, electrode design, and stimulation parameters^17^. Stimulation parameters vary among studies, with different stimulation frequencies, pulse widths, polarities, stimulation times, and utilized pulse types^13^. However, no standardized stimulation parameters elicit optimum efficacy and safety^18^. Therefore, regarding the safety aspect of taVNS, the method of monitoring and assessment methods, the frequency of adverse events, and their relationship with clinical variables have not been established. This review aimed to (1) systematically analyze study characteristics and AE assessment, (2) characterize and analyze possible AEs and their incidence, (3) search for predictable risk factors, (4) analyze AEs by severity, and (5) suggest an evidence-based taVNS adverse events questionnaire for safety monitoring during taVNS.

## Methods and analysis

### Literature search

This systematic review was registered at PROSPERO (registration number 340559), and it was conducted according to the guidelines of the Preferred Reporting Items for Systematic Reviews and Meta-Analyses–PRISMA^19^ and the Cochrane Handbook for Systematic Reviews^20^. We searched for articles published through April 7, 2022, in Medline, Embase, Web of Science, Cochrane, and Lilacs databases, using the following search terms: (("Transcutaneous vagal nerve stimulation") OR ("Transcutaneous vagus nerve stimulation") OR ("Auricular vagal nerve stimulation") OR ("Auricular vagus nerve stimulation") OR (tVNS) OR (taVNS) OR (aVNS)).

### Inclusion and exclusion criteria

The following criteria were adopted for eligibility: (1) articles published in English; and (2) articles that reported the effects of taVNS in humans. Therefore, we excluded the following articles: (1) animal studies; (2) review articles; (3) articles reporting duplicate data or data extracted from original articles; (4) abstracts lacking full text; and (5) articles addressing other brain stimulation techniques.

### Data extraction

Search findings were imported into Endnote Version X9, and uploaded to the Covidence system, where duplicate articles were removed. To independent reviewers (AM and AYK) performed the title and abstract screening process. Two reviewers then read the full text to assess their eligibility for inclusion. During the review process, studies were excluded under consensus if they did not meet our criteria, and a third reviewer was consulted to resolve any conflicts (FF). Out of 824 articles, 175 articles were included for review, and data from each article were sorted out for analysis. Four reviewers independently (ACLG, AM, AYK, and PdSM) extracted data using a predefined data extraction template in Excel. Any discrepancies were resolved by consensus, with the corresponding author (FF) consulted when necessary. The reviewers developed a structured checklist that included the following items: (1) Study characteristics: first author, title, journal, year of publication, country, etc.; (2) Trial characteristics: design, randomization method, blinding, etc.; (3) Participant characteristics: sample size, age, sex, comorbidity (healthy vs. disease), etc.; (4) Intervention characteristics: device type, interventional protocol (number of sessions, stimulus frequency, intensity, pulse, target area, duration, etc.), and control group (no vs. yes); (5) Outcomes: primary outcome, secondary outcome, and main conclusions; (6) Assessment method of adverse events; (7) Adverse and severe adverse events. The original authors were contacted for any missing data. Discrepancies were resolved through them through team discussions.

### Quantitative analysis

#### Comparison of study characteristics

For this analysis, we divided the studies into three groups: (1) studies not reporting AEs; (2) studies reporting no AEs, and (3) studies reporting at least 1 AE. Continuous variables were reported as mean and standard deviations and compared using one-way analysis of variance. Categorical variables were reported as absolute numbers and percentages and were compared by Fisher's exact test. We performed Bonferroni corrections for multiple comparisons to interpret the results.

#### Analysis of the effects of active taVNS on adverse events

Furthermore, a quantitative analysis was performed on the clinical trials reporting the number of subjects with at least one adverse event or the intensity of the adverse events in the active taVNS and the control groups.

For the studies with the number of subjects who reported at least one adverse event, we performed a random-effects meta-analysis to calculate the pooled risk differences and the 95% confidence interval (CI) based on the Mantel–Haenszel method. We calculated specifically the risk differences developing in ear pain, dizziness, skin redness, and headache using the same approach. In this analysis, the command "metabin" from the "meta" package in RStudio was used^21^.

In the studies reporting the intensity of adverse events in both groups, a random-effects meta-analysis was performed to calculate the pooled standard mean difference and 95% CI, based on the inverse variance method, of intensity in headaches, nausea, unpleasant feelings, neck pain, changes in concentration, stinging sensation, muscle contraction, burning sensation, dizziness, skin irritation, and fluctuation feeling. For this analysis, the command "metacont" from the "meta" package in RStudio was used^21^.

#### Overall incidence in the population exposed to active taVNS

Aiming to detect the most common adverse events related to taVNS, we analyzed all the studies that documented the number of subjects exposed to active taVNS, the number of subjects that reported at least one event, the duration of the taVNS sessions, and the number of days that a session was delivered. The product of the duration of the session and the number of days that the session was delivered was called cumulative exposure time (if there were more than one session per day, the time was multiplied by the number of sessions). The overall incidence rate was calculated for the total number of adverse events and for each type of adverse event reported on a 100,000 person-minutes-days scale. To perform the meta-analytic incidence, random-effects and the inverse variance method were used to the pooled incidence and the 95% confidence intervals. We used the command “metarate” from the “meta” package in RStudio in this analysis^21^.

#### Analysis of stimulation parameters effects on the adverse events per person

We modeled a linear regression to identify associated the factors with the development of adverse events in subjects who underwent active taVNS. To control for the number of subjects in each study, we created a variable, the number of adverse events per person, which consisted of the number of patients experiencing adverse events receiving active taVNS in each study over the total number of subjects receiving active taVNS per study. To select the covariates for the model, we performed univariate regressions, accepting a significance of *p* < 0.25 for their inclusion in the multivariate model. A "purposeful selection" method was used to create the multivariate model; covariates that were not statistically or clinically significant as well as confounders were removed from the model^22^. Covariates were considered confounders if they changed the beta coefficient of the dependent variable by ≥ 10%. Moreover, biologically plausible variables, such as age and the prevalence of female subjects, were included in the multivariate model as well.

Studies were included in the linear regression analysis if they had an active taVNS group (i.e., one arm or two-arm studies) and reported the number of patients experiencing adverse events in said group. All analyses were performed using RStudio version 2022.2.2.485^23^.

### Reporting of the severe adverse events and the relatedness with taVNS

All the adverse events reported as severe by the authors were analyzed individually for their severity using the Common Terminology Criteria for Adverse Events (CTCAE). The grades were 1–5, being in general (1) mild symptoms; (2) moderate; (3) severe but not life-threatening; (4) life-threatening consequences; and (5) death related to adverse events. For this review, we considered grades 3–5 as severe^24^.

To determine if the adverse events classified as severe were related to the taVNS, we used the nine criteria of causality proposed by Bradford Hill. According to these criteria, a relationship between two variables, to be defined as causation, must be analyzed regarding the: (1) association strength; (2) consistency; (3) specificity; (4) temporality; (5) biological gradient; (6) plausibility; (7) coherence; (8) experiment; and (9) analogy^25^.

### Quality assessment

The risk of bias was assessed in controlled studies, either parallel arm or cross-over randomized controlled trials (RCTs), using Cochrane Collaboration’s tool (Risk of Bias Tool 2.0)^26^ concerning its specific domains.

The non-randomized studies (case reports and one arm studies) were inherit classified as a high risk of bias by our research team, since these studies do not have randomization, blinding, and a control group.

## Results

We retrieved 824 articles using the search term previously mentioned, and after excluding studies according to our selection criteria, 174 articles remained with a total of 177 experiments (seven articles presented two experiments and two articles presented duplicated data) remained. Throughout this paper, the term “study” refers to the *experiment* and not the *article*. Seventy-nine (44.63%) studies reported the presence or absence of adverse events, while 98 (55.37%) studies did not. Only 44 (24.86%) studies reported at least one adverse event occurred. The flowchart detailing the study selection is provided in supplementary material 1.

### Studies that did versus did not report the presence/absence of AEs

We evaluated 177 studies that assessed 6322 subjects with a mean age of 34.99 (S.D. = 15.15) years and 53.51% of the subjects were female. Most studies were RCT with either a cross-over or parallel design. Of them, 55.37% (98/177) of studies did not mention the presence or absence of any AEs. Most studies also included a control group. Regarding taVNS parameters, most studies in all three groups reported their characteristics (Table 1).

**Table 1:** Study characteristics and taVNS parameters of studies not reporting AEs, studies reporting no AEs, and studies reporting at least one AE.

	Studies not reporting AEs (N = 98)	Studies reporting no AEs (N = 35)	Studies reporting at least 1 AE (N = 44)	*p*-value
Gender (average female %)	50.44	54.69	55.40	0.108
Age (mean ± SD)	33.72 ± 14.17	35 ± 16.27	37.49 ± 16.20	0.207
Sample size (mean ± SD)	35.20 ± 23.43	26.08 ± 27.57	44.52 ± 40.54	0.243
**Population**				
Healthy	67	19	13	0.001*
Non-healthy	31	16	31	
**Study design**				
Parallel	26	11	22	–
Cross-over	52	15	9	
One arm	13	7	12	
Pilot study	1	1	0	
Case report	3	1	1	
**Control group?**				0.214
Yes	71	20	28	
No	27	15	16	
**Duration**				0.039
0–60 min	71	22	25	
> 60 min	19	11	18	
**Number of sessions**				
1–2 sessions	70	19	14	< 0.001*
Repeated sessions	27	14	30	
**Intensity**				
Defined	25	10	10	0.791
Adjustable	66	21	30	
**Frequency**				
1–20 Hz	12	6	6	0.780
20–30 Hz	82	28	35	
**Pulse width**				
0–250 µs	50	13	17	0.062
300 µs >	25	18	12	
**Stimulation side**				
Unilateral	70	31	29	0.004*
Bilateral	8	0	10	

*Significant after Bonferroni's correction (*p* < 0.004).

AE: adverse event; SD: standard deviation; Hz: Hertz; µs: micro-seconds.

When comparing studies reporting no AEs, or at least one AE vs. non-reporting AEs, we observed significant differences in population type, number of sessions, and stimulation side. A significantly higher number of studies evaluated taVNS in healthy populations and unilaterally without mentioning AEs, versus reporting no AEs or at least one AE. Moreover, the study group not reporting AE had significantly more studies with 1–2 sessions when compared with the studies reporting no AE or at least one AE.

We contacted the corresponding authors of the studies that did not report AEs to inquire about any relevant information from their trials; a total of 14 authors replied. Of them, 11 answered that their participants did not report any AEs and all the information was included in the published articles, while four studies reported mild adverse events, including discomfort with the ear clip (n = 1), skin irritation (n = 1), headache (n = 1), and annoying sensation of the stimulation (n = 1). These studies were included in their respective groups in the supplementary material 2; the results were similar, and these data were not used in the subsequent analyses.

### Studies that reported AE, device analysis, and assessment method

A total of 76 articles (79 studies) that reported adverse events were used for the data extraction. The characteristics of these studies are summarized in Table 2. The most common study populations were healthy (40.51%), epilepsy (15.19%), tinnitus (7.60%), depression (6.33%), stroke (6.33%), and migraine (2.53%). Three studies were of pediatric populations, one each of epilepsy, nephrotic syndrome, and infant oral feeding dysfunction. There were 33 parallel, 24 cross-over, 19 one arm, one pilot study, and two case studies.

**Table 2:** Characteristics of included studies that report adverse events according to study population.

Study	Design	Region	N	Age (years)	Gender (F/M)	Number of sessions	Duration (minutes)	Intensity (mA)	Frequency (Hz)	Pulse width (µs)	Device	Stimulation side	Active stimulation site	Sham stimulation site
**Healthy**														
Busch 2012	Cross-over	Germany	48	23.3 ± 2.1	24/24	1	60	0.25–10	25	250	STV02, Cerbomed	Left	Cymba Conchae	Cymba conchae
Capone 2014	Cross-over	Italy	10	30.2 ± 3.6	7/3	1	60	n/a	20	300	Twister, EBM & Ag–AgCl electrodes	Left	External acoustic meatus	Earlobe
Jacobs 2015	Cross-over	The Netherlands	30	60.57 ± 2.54	15/15	n/a	n/a	5	8	200	TENSTem dental, Schwa-medico	Left	External acoustic meatus of the inner side of the tragus	Earlobe
Sellaro 2015	Parallel	The Netherlands	45	20.65 ± 1.7	40/5	1	75	0.5	25	200–300	CM02, Cerbomed	Left	Outer auditory canal	Earlobe
Steenbergen 2015	RCT, parallel	The Netherlands	30	19.8	26/4	1	25	0.5	25	200–300	CM02, Cerbomed	Left	Outer ear canal	Earlobe
Burger 2016	Parallel	The Netherlands	31	21.5	24/7	1	n/a	0.5	25	n/a	NEMOS, Cerbomed	Left	Cymba conchae	Earlobe
FRØKJÆR 2016	Cross-over	Denmark	18	51.6	8/10	1	60	0.1–10	30	250	NEMOS, Cerbomed	Left	Cymba conchae	Earlobe
Sellaro 2017	Cross-over	The Netherlands	24	20.71 ± 2.35	15/9	2	20	0.5	25	200–300	NEMOS, Cerbomed	Left	Cymba conchae	Earlobe
Badran 2018 (Trial 1)	Cross-over	USA	15	26.5 ± 4.99	7/8	1	9	200% of the perceived threshold	1–25	100–500	DS7, Digimeter	Left	Inner side of tragus	Earlobe
Badran 2018 (Trial 2)	Cross-over	USA	20	25.65 ± 5.53	10/10	1	10	200% of the perceived threshold	10–25	500	DS7, Digimeter	Left	Inner side of tragus	Earlobe
Burger 2018	RCT, parallel	The Netherlands	85	21.01 ± 1.87	71/14	1	25	0.5	25	250	NEMOS, Cerbomed	Left	Cymba Conchae	Earlobe
Colzato 2018	Cross-over	The Netherlands	32	21.34 (18–28)	22/10	1	50	0.5	25	200–300	NEMOS, Cerbomed	Left	Outer auditory canal	Earlobe
Fischer 2018	Cross-over	Germany	21	20.3 ± 1.4	18/3	1	36	0.4–4.8	25	200–300	CMO2, Cerbomed	Left	Cymba conchae	Earlobe
Jongkees 2018	Cross-over	Germany	40	22.4	32/8	1	15	0.5	25	200–300	CMO2, Cerbomed	Left	Outer auditory canal	Earlobe
Ventura-Bort 2018	Cross-over	Germany	21	20.3 ± 1.4	18/3	1	28	Adjustable	25	200–300	NEMOS, Cerbomed	Left	Cymba conchae	Earlobe
Sclocco 2019	One arm	USA	16	27.0 ± 6.6	9/7	1	8	n/a	25	450	Grass S88x stimulator, Astro-Med	Left	Cymba Conchae	None
Warren 2019	Cross-over	Germany	24	22.6	18/6	1	20	0.5	25	200–300	NEMOS, Cerbomed	Left	Cymba conchae	Earlobe
Giraudier 2020	Parallel	Germany	60	23.39 ± 4.67	47/13	1	23	0.5–3.5	25	200–300	Cerbomed GmbH	Left	Cymba conchae	Earlobe
Maraver 2020	Cross-over	The Netherlands	43	20.00 ± 2.34	39/4	2	60	0.5	25	200–300	NEMOS, Cerbomed	Left	Cymba conchae	Earlobe
Pihlaja 2020	Cross-over	Finland	25	25.5 ± 4.8	16/9	1	n/a	1.5–3.2	30	250	Salustim, Helsinki Ear Institute	Left	Tragus	Earlobe
Ricci 2020	Cross-over	Italy	8	30.5 ± 6.02	0/8	1	60	0–8	30	500	Twister, EBM & Ag–AgCl electrodes	Left	External acoustic meatus	Earlobe
Sclocco 2020	Cross-over	USA	30	29.0 ± 9.8	17/13	4	8.55	Adjustable	2, 10, 25, 100	300	UROstim, Schwa-medico	Left	Cymba conchae	Cymba conchae
Sharon 2020	Cross-over	Israel	25	28.08 ± 5.84	0/25	1	5	Adjustable	25	200–300	NEMOS, Cerbomed	Left	Cymba Conchae	Earlobe
Thakkar 2020	Parallel	USA	37	21.05	27/10	n/a	30	Adjustable	5	200	n/a	Left	Cymba conchae	Earlobe and cymba conchae
D’Agostini 2021	Parallel	Belgium	71	23.09 ± 3.62	55/16	1	40	0.5	25	250	NEMOS, Cerbomed	Left	Cymba conchae	Earlobe
D’Agostini 2022	Cross-over	Belgium	66	21.74 ± 2.60	n/a	1	50–55	Adjustable	25	250	NEMOS, Cerbomed	Left	Cymba conchae	Earlobe
Gurtubay 2021	One arm	Spain	26	22–59	13/13	1	4–8	2.4*	1	500	Custom Ag–AgCl electrodes	Left	Cymba and cavum, cymba and earlobe	n/a
Machetanz 2021	Quasi-randomized, parallel	Germany	13	24.0 ± 3.0	8/5	1	1.5	0.05–0.769	25	100, 260, 500	STG-4000 series, Harvard Bioscience Inc	Bilateral	Cymba conchae, cavum conchae, outer tragus, inner tragus, crus helicis, fossa triangularis (each separately)	n/a
Sun 2021	Cross-over	China	46	20.39 ± 1.95	25/21	3	15	Adjustable	25	500	XD-Kerfun BS-VNS-001, Intelligent Non-invasive Neuromodulation Technology and Transformation Joint Laboratory, Xidian University	Left	Inner side of tragus	Earlobe
Széles 2021	Cross-over	Austria	9	50.7 ± 7.2	4/5	4	44	n/a	1	500	PrimeStim, TUWien	Right	Cymba and cavum concha, antihelix crura	n/a
Geng 2022 (Study 1)	Cross-over	China	14	23.42 ± 1.29	6/8	1	5	n/a	20	250	The Parasym, Parasym	Left	Tragus	Earlobe
Geng 2022 (Study 2)	One arm	China	20	21.51 ± 2.12	9/11	1	20	n/a	5 or 20	50 or 250	The Parasym, Parasym	Left	Tragus	Earlobe
Konjusha 2022	Cross-over	Germany	45	23.57 ± 0.51	37/8	1	20	0.5	25	200–300	CM02, Cerbomed	Left	Outer ear	Earlobe
**Epilepsy**														
Stefan 2012	One arm	Germany	10	37.7 (18–55)	6/4	810	60	n/a	10	300	n/a	Left	Auricular branch of the vagus nerve	n/a
He 2013**	One arm	China	14	7.64	3/11	216	30	n/a	20	n/a	TENS-200, Suzhou	Bilateral	Conchae cavum and cymba conchae	n/a
Aihua 2014	RCT, parallel	China	60	34.5 (26.5–41.3) 29 (24.5–42)	n/a	1095	20	adjustable	20	200	TENS-200, Suzhou	Bilateral	Ramsay-hunt zone	Earlobe
Peijing 2014	One arm	China	50	25.2 ± 13.1	20/30	336	30	1	20–30	n/a	TENS, Suzhou	n/a	Triangular fossa of the auricle	n/a
Rong 2015	Parallel	China	144	n/a	n/a	336	30	1	20–30	< 100	TENS, Suzhou	Bilateral	Triangular fossa of the outer ear canal	n/a
Bauer 2016	RCT, parallel	Germany	76	38.8	45/31	140	240	Adjustable	25, 1	250	NEMOS, Cerbomed	Left	Cymba conchae	Cymba conchae
Barbella 2018	Quasi-randomized, crossover-like	Italy	20	38.6	10/10	180–540	60	0.6–0.8	n/a	n/a	n/a	n/a	n/a	n/a
Song 2018	Parallel	China	52	53.7	19/33	8	30	n/a	1	n/a	NEMOSR, Cerbomed	Bilateral	Cymba conchae	n/a
Liu 2018	One arm	China	17	27 ± 9.4	7/10	540	20	Adjustable	10	200	TENS-sm, Suzhou	bilateral	Conchae cavum and cymba conchae	n/a
Von Wrede 2019	Case report	Germany	1	24	1/0	380	240	1.5	n/a	n/a	CM02, Cerbomed	n/a	n/a	n/a
Sabers 2021	One arm	Denmark, Norway, The Netherlands, Belgium	37	40& median	23/14	180, 360	240	0.5–5	25	250	NEMOS, Cerbomed	Left	Cymba conchae	n/a
Von Wrede 2021	One arm	Germany	14	41&	8/6	1	60	Adjustable	25	n/a	NEMOS, Cerbomed	Left	Cymba conchae	None
**Tinnitus**														
Kreuzer 2012	One arm	Germany	24	59 ± 10.7	14/10	5–43	300	n/a	25	n/a	CM02, Cerbomed	Left	n/a	n/a
LEHTIMÄKI 2013	Parallel	Finland	10	n/a	n/a	7	45–60	Adjustable	25	n/a	Tinnoff pulse generator, Tinnoff Inc	Left	Tragus	n/a
Kreuzer 2014 (Phase 2)	One arm	Germany	50	n/a	n/a	96	240–360	0.1–10	25	n/a	**Phase 1** CM02, Cerbomed **Phase 2** NEMOS, Cerbomed	n/a	n/a	n/a
Shim 2015	One arm	South Korea	30	58.47 ± 15.85	9/21	10	30	1–10	25	200	TENS eco2, Schwa-medico	Left	Auricular conchae	n/a
Suk 2018	One arm	South Korea	24	44.58	8/16	4	48	Adjustable	30	200	ES-420, Ito Company Ltd	n/a	Cavum, cymba, outer tragus	n/a
Ylikoski 2020	One arm	Finland	171	49 (7–84)	67/104	5	60–90	Adjustable	25	250	Salustim, Helsinki Ear Institute	Left	tragus	n/a
**Depression**														
Hein 2013 (Study 1)	RCT, Parallel	Germany	22	45.8	13/9	10	15	Adjustable	1.5	n/a	NET-200, Auri-Stim Medical	Bilateral	Outer ear canal	Outer ear canal
Hein 2013 (Study 2)	RCT, Parallel	Germany	15	48.1	9/6	20, 10	15	0.13	1.5	n/a	NET-1000, Auri-Stim Medical	Bilateral	Outer ear canal	Outer ear canal
Rong 2016	RCT, parallel	China	160	41.99	117/43	168	30	Adjustable	20	200	TENS-200, Suzhou	n/a	Cymba conchae	Superior scapha
Evensen 2021	One arm	Denmark	20	49.4 ± 11.2	13/7	28	240	0.1–5	25	n/a	NEMOS, Cerbomed	Left	Auricular tract	n/a
Kaczmarczyk 2021	One arm	Poland	5	53.6	3/2	144	120	0.5–5	25	n/a	NEMOS, Cerbomed	n/a	n/a	n/a
**Stroke**														
Capone 2017	Parallel	Italy	12	54.65	5/7	10	60	Adjustable	20	300	Twister, EBM & Ag–AgCl electrodes	Left	External acoustic meatus	Earlobe
Redgrave 2018	One arm	United Kingdom	13	64.5 ± 6	3/10	18	60	1–3.2	25	100	NEMOS Cerbomed	Left	Cymba conchae	n/a
Wu 2020	Parallel	China	21	63.16	8/13	15	30	Adjustable	20	300	HD-1A, Bohua	Left	Cymba conchae	Cymba conchae
Chang 2021	Parallel	USA	36	59.02 ± 1.98	18/18	9	60	0–5	30	300	Custom conductive silicone electrodes with wireless Bluetooth control	Left	Cymba conchae	Cymba conchae
Li 2022	Parallel	China	60	68.75	31/29	20	20	Adjustable	20	300	TENS, Suzhou	Left	Cymba conchae	Cymba conchae
**Migraine**														
Straube 2015	RCT, parallel	Germany	46	41.55	39/7	90	240	Adjustable	25, 1	250	NEMOS Cerbomed	Left	Cymba conchae	n/a
Cao 2021	RCT, cross-over	USA	24	31.33 ± 1.55	21/3	2	8	Adjustable	20, 1	200	SDZ IIB, Suzhou	Left	Cymba and cavum concha	Cymba and cavum concha
**Bruxism**														
Polini 2022	One arm	Italy	10	37.2 (19–52)	7/3	4	30	n/a	n/a	n/a	Custom device	Bilateral	Cavum conchae, external auditory meatus, internal tragus	n/a
**Chronic fatigue syndrome**														
Early 2018	Case report	USA	1	58	1/0	420	240	n/a	n/a	n/a	n/a	Left	Cymba conchae	n/a
**Erosive hand osteoarthritis**														
Courties 2022	Pilot study	France	20	69 (66.7–73.2)	15/3	28	60	0–15	25	50	TENS eco2, Schwa-medico	Left	Cymba conchae	n/a
**Fibromyalgia**														
Kutlu 2020	Parallel	Turkey	60	39.02	60/0	20	30	n/a	10	500	TENS device (not specified)	Bilateral	Inner and rear surfaces of tragus and conchae	n/a
**Functional dyspepsia**														
Zhu 2021	Parallel	China and USA	36	44.2	25/11	14	120	0.5–1.5	25	n/a	SNM-FDC01, Ningbo Maida Medical Device	Bilateral	Cymba conchae	n/a
**Generalized anxiety disorder**														
Burger 2019	Parallel	The Netherlands	97	n/a	78/19	1	15	0.5	25	250	NEMOS, Cerbomed	Left	Cymba conchae	Earlobe
**Heart failure**														
Stavrakis 2022	Parallel	USA	52	69.8	32/20	90	60	Adjustable	20	200	The Parasym, Parasym	n/a	Tragus	Earlobe
**Impaired glucose tolerance**														
Huang 2014	Parallel	China	72	54.4 ± 7.4	51/21	294	20	1	20	< 1000	TENS-200, Suzhou	n/a	Cymba conchae	Superior scapha
**Insomnia**														
Jiao 2020	RCT, parallel	China	72	48.5	60/12	40	30	Adjustable	20	n/a	SDZ-IIB, Suzhou	Bilateral	Auricular concha	n/a
**Left-ventricular strain**														
Tran 2018	RCT, cross-over	USA	24	68.3 ± 1.2	11/13	2	60	Adjustable	20	200	The Parasym, Parasym	Left	Tragus	Tragus
**Open laparotomy patients**														
Hong 2018	One arm	Germany	14	57.6 ± 10.5	8/6	1	10	10	25	250	Transcutaneous, bipolar stimulation probe (Stimulationssonde 522,015, Inomed)	Right	Cymba conchae	n/a
**Pediatric nephrotic syndrome**														
Merchant 2022	Pilot study	USA	7	9.5 ± 4.2	3/4	182	5	Adjustable	30	300	TENS 7000, Roscoe Medical	Left	Cymba conchae	n/a
**Pediatric oral feeding dysfunction**														
Bandran 2020	One arm	USA	14	45 ± 5 +	9/5	14	30	Adjustable	25	500	DS7AH, Digitimer	Left	Tragus	n/a
**Prader-Willi syndrome**														
Manning 2019	One arm	United Kingdom	5	26.4 ± 7.36	2/3	180, 360	240	0.1–5	25	250	NEMOS, Cerbomed	Left	Cymba conchae	n/a
**Schizophrenia**														
Hasan 2015	RCT, parallel	Germany	17	36.5	9/8	182, 96	289#	0.1–10	25	250	CM02, Cerbomed	Left	Outer ear canal	Outer ear canal
**Systemic lupus erythematosus**														
Aranow 2020	Parallel	USA	18	45.7 ± 11.7	18/0	4	5	Adjustable	30	300	TENS 7000, Roscoe Medical	Left	Cymba conchae	Earlobe
**Vegetative state**														
Hakon 2020	One arm	Denmark	5	51.8 ± 24.4	1/4	56	240	0.5, 1	25	250	NEMOS, Cerbomed	Left	Cymba conchae	n/a

*mean mA;**pediatric population;&median; + days; #mean duration of 4 groups.

AE: adverse event; SD: standard deviation; Hz: Hertz; µs: micro-seconds; n/a: non-applicable; RCT: randomized clinical trial; F/M: female/male.

The types of devices and electrodes used for the stimulation varied significantly. Of the 79 studies that were reviewed, 41 studies (51.3%) used specific commercial devices designed for taVNS. The most common was NEMOS by Cerbomed (Erlangen, Germany) used in 22 studies (27.5%), which consisted of two titanium electrodes positioned a top of a silicon earplug designed to stimulate the left cymba concha. Seven studies (8.8%) use a specific clip electrode inserted into the left tragus^27^. The NET-1000 and NET-2000 (Auri-Stim Medical, CO, USA) used four 3 mm electrodes placed crosswise to stimulate the bilateral outer ear canals^28^, while 35 studies (43.8%) used off-label custom devices, and four studies (5.0%) did not specify the equipment used. The majority of the studies (64 studies, 81.01%) stimulated the left side, while five studies (6.3%) stimulated the right side and 10 studies (12.6%) stimulated both sides. Most studies used stimulation sites within the cymba concha, cavum concha, tragus, and external acoustic meatus. The earlobe was the most common site for sham stimulation. Two studies used 1 Hz stimulation as an active control for comparison with 25 Hz taVNS stimulation, both at the left cymba concha^29^.

The methods of AEs assessment varied among the studies. Thirty-four studies (43.03%) did not specify the method of assessment but reported only AEs results. Other studies used qualitative assessment methods such as patient interviews, diaries, self-reports, and clinician objective monitoring. Notably, 18 studies used a quantitative scale in which participants were asked to rate potential side effects such as headache, neck pain, dizziness, tiredness, nausea, tingling sensations, skin irritations at the ear, and concentration, and mood changes on a point scale^30^. All of these studies had a control group, and 6 studies (33.3%) reported that there was no significant difference versus the sham group participants (Table 2).

### Effects of active taVNS on the development of adverse events

To assess the effects of active taVNS on the development of adverse events, we performed a quantitative analysis on the clinical trials that reported this information in their active and control groups.

#### Analysis of subjects reporting at least one adverse event

A total of 35 studies included in this review reported the number of subjects who developed at least one adverse event. This analysis gathered all available data of this review regarding the number of patients reporting AEs in the active and in the control groups: pooled data were obtained from 1708 patients.

##### Risk differences in development of any adverse events versus control

**Figure 1:**
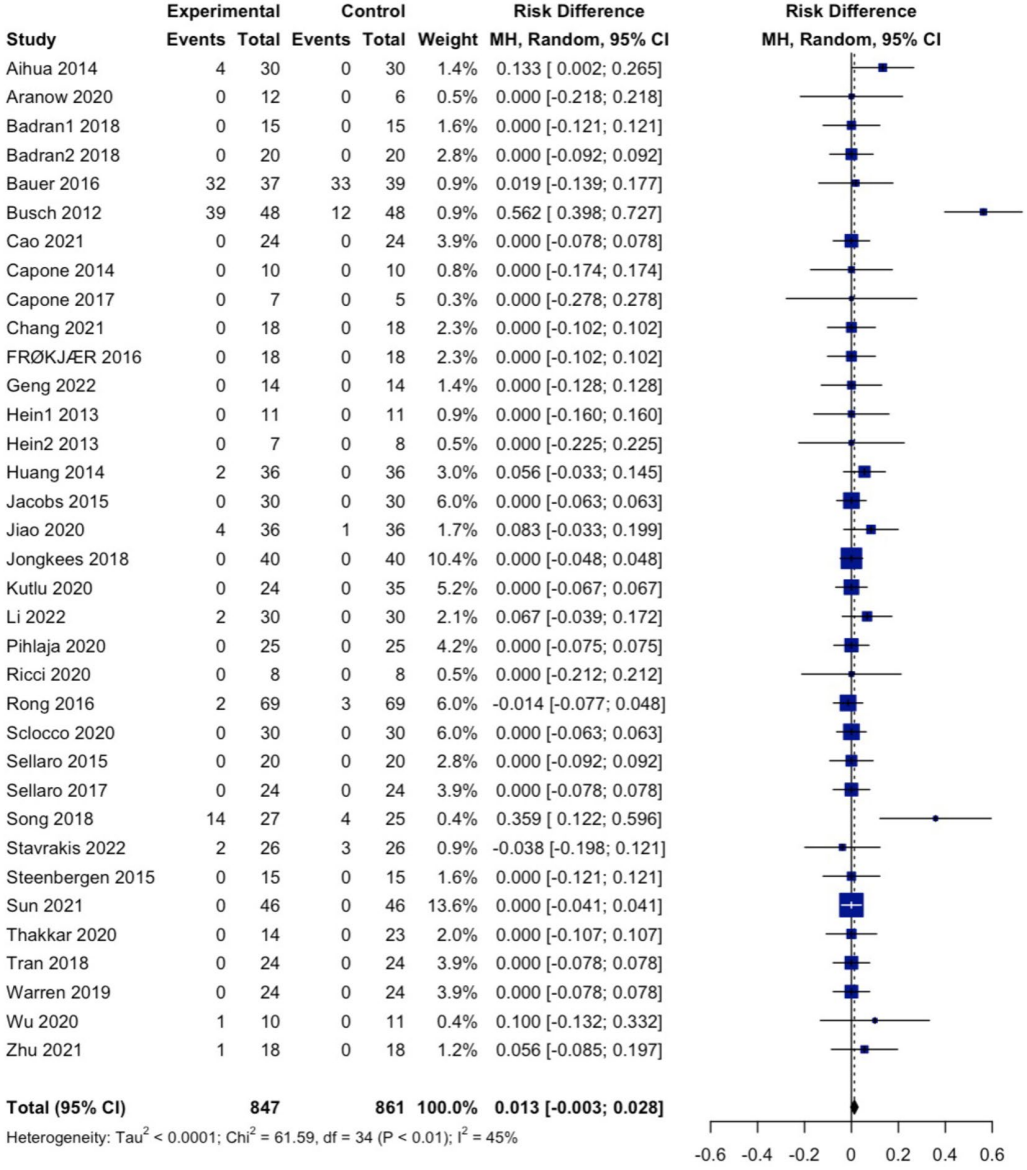
Forest plot of the studies that reported the number of subjects reporting at least one adverse event in the active and sham groups.

There were no differences in the risk of developing an adverse event between the active taVNS and controls (RD 0.013, 95% CI − 0.003 to 0.028, I^2^ = 45%). A forest plot is shown in Fig. 1.

##### Differences in develop ear pain, dizziness, headache, and skin redness in comparison to control

Our analysis showed no risk differences in developing specific AE as ear pain, dizziness, skin redness, and headache. All of them presented a pooled risk difference of 0.00 (95% CI − 0.01 to 0.01, I^2^ = 0%), detailed information of these analyzes is provided in the supplementary material 3.

#### Analysis of differences in intensity of the adverse events

Nine studies included in our review reported the adverse events on different scales with different ranges representing the intensity of each symptom. Depending on the adverse event reported, there was a different number of studies and subjects included in the analysis because of the availability of the data.

From the adverse events available to analyze, our review did not detect intensity differences in any of them. Headaches (n = 567, SMD − 0.21, 95% CI -0.46 to 0.05, I^2^ = 56%), nausea (n = 567, SMD 0.14, 95% CI -0.08 to 0.36, I^2^ = 40%), unpleasant feeling (n = 567, SMD 0.05, 95% CI -0.20 to 0.29, I^2^ = 50%), neck pain (n = 481, SMD 0.06, 95% CI − 0.52 to 0.64, I^2^ = 89%), changes in concentration (n = 391, SMD 0.17, 95% CI − 0.33 to 0.68, I^2^ = 81%), stinging sensation (n = 368, SMD 0.66, 95% CI − 0.08 to 1.39, I^2^ = 91%), muscle contraction (n = 368, SMD 0.14, 95% CI − 0.09 to 0.37, I^2^ = 25%), burning sensation (n = 365, SMD 0.93, 95% CI − 0.16 to 2.02, I^2^ = 95%), dizziness (n = 192, SMD − 0.28, 95% CI − 0.61 to 0.05, I^2^ = 25%), skin irritation (n = 192, SMD 0.82, 95% CI -0.38 to 2.03, I^2^ = 91%), and fluctuation feeling (n = 144, SMD 0.43, 95% CI − 0.35 to 1.20, I^2^ = 80%). The forest plots and further information is provided in the supplementary material 4.

### Overall incidence of adverse events in taVNS

From the 80 studies reporting the presence or absence of adverse events, 56 studies were included to calculate the cumulative incidence of the events described in the literature and define the most common adverse events related to taVNS. The 24 studies not included in this analysis did not clearly report the cumulative time of the stimulation, the total number of subjects exposed to taVNS, or did not report the number of subjects that developed the events. From 1330 patients analyzed, there were 167 that reported at least one adverse event. The total cumulative exposure time was 484,812 min-days of stimulation. The total cumulative incidence of adverse events was 12.84 events/ 100,000 person-minutes-days (95% CI 6.65 to 24.80). Based on our analysis, the three most common adverse events in consideration to the cumulative exposure time to taVNS were ear pain, headache, and tingling. The full order of the most common adverse events and the detailed information of the cumulative incidences can be found on Table 3.

**Table 3:** Meta-analytic pooled incidence rates (events/100,000 person-minutes-days of stimulation) for the total and each adverse event in patients exposed to taVNS.

Type of adverse event	Number of events	Pooled incidence rate/100,000 person-minutes-days (95% CI)
Ear pain	17	7.35 (3.61;14.96)
Headache	21	7.01 (3.49;14.06)
Tingling	10	6.91 (3.28;14.55)
Dizziness	14	6.89 (3.42;13.87)
Skin redness	8	6.89 (3.33;14.26)
Fatigue	8	6.82 (3.33;13.98)
Prickling	15	6.77 (3.18;14.43)
Pressure	9	6.75 (3.20;14.27)
Itching	11	6.75 (3.18;14.32)
Unpleasant feeling	5	6.61 (3.16;13.81)
Nausea	5	6.55 (3.18;13.49)
Nasopharyngitis	10	6.51 (3.19;13.28)
Vertigo	5	6.48 (3.15;13.29)
Hospitalization	3	6.42 (3.11;13.23)
Insomnia	2	6.38 (3.08;13.20)
Drowsiness	3	6.37 (3.09;13.11)
Mood change	2	6.36 (3.08;13.15)
Tinnitus	2	6.32 (3.05;13.08)
Conjunctivitis	1	6.31 (3.04;13.10)
Scotoma	1	6.31 (3.04;13.10)
Floating body	1	6.31 (3.04;13.10)
Desadaptation	1	6.31 (3.04;13.10)
Hand pain	1	6.31 (3.04;13.10)
Head full	1	6.31 (3.04;13.09)
Diarrhea	2	6.30 (3.05;13.03)
Hearing loss	1	6.29 (3.04;13.06)
Neck pain	1	6.29 (3.03;13.04)
Vibration	1	6.29 (3.03;13.04)
Crimpling sensations	1	6.29 (3.03;13.04)
Flashbacks	1	6.29 (3.03;13.04)
Feeling of flaccidness	1	6.29 (3.03;13.04)
Stinging	2	6.27 (3.03;12.99)
Hoarseness	1	6.27 (3.02;13.01)
Obstipation	1	6.27 (3.02;13.01)
Skin irritation	2	6.26 (3.02;12.97)
Total	167	12.84 (6.65;24.80)
Total number of subjects	1330	
Total cumulative exposure time (minutes of stimulation x days of stimulation)	484,812 min-days	

CI: confidence interval.

### Effects of parameters of stimulation on the AE

When analyzing for the relationships between taVNS parameters and the number of AEs per person receiving taVNS in the included studies, univariate analyses conveyed a significant association between the number of adverse events per person and duration of taVNS, number of sessions, and type of devices performing taVNS. Specific taVNS devices were shown to be significantly more likely to lead to AEs than off-label devices (ß: 0.149, 95% CI: − 0.010 to 0.289, *p*-value: 0.036). Repeated sessions and sessions lasting 60 min or more are also shown to be more likely to lead to AEs (ß: 0.126, 95% CI: 0.019 to 0.232, *p*-value: 0.022; and ß:0.151, 95% CI: 0.049 to 0.252, *p*-value: 0.004, respectively).

Furthermore, in the multivariate analysis, age, and higher prevalence of female subjects, proved not to be significant influences on the likelihood of a subject having an AE after taVNS across the assessed studies (ß: − 4.73, 95% CI: − 0.00003 to 0.00002, *p*-value: 0.727; and ß: 0.053, 95% CI: − 0.113 to 0.220, *p*-value: 0.520, respectively). When evaluating the inclusion of taVNS parameter variables, two of them proved to be significant on their own; duration of stimulation (ß: 0.285, 95% CI: 0.122 to 0.448, *p*-value: 0.001) and type of devices (ß: 0.061, 95% CI: 0.045 to 0.381, *p*-value: 0.014). However, when both were included in the model together, none of them proved to be significant. Nonetheless, these two variables proved to be colinear, thus the final multivariate analysis conveys the best fit model between the two, the one in which duration of stimulation was significant (Table 4).

**Table 4:** Univariate and multivariate regression analysis of taVNS parameters associated with the number of AEs reported by patients undergoing active taVNS in the included studies.

	Beta-coefficient	95% CI	*p*-value
**Univariate analysis**			
Age	0.001	− 0.002 to 0.006	0.422
Gender	0.275	− 0.037 to 0.588	0.082
Number of subjects	0.001	− 0.0003 to 0.004	0.099
Duration	0.151	0.049 to 0.252	0.004
Number of sessions	0.126	0.019 to 0.232	0.022
Intensity	0.070	− 0.067 to 0.208	0.312
Frequency	0.083	− 0.032 to 0.199	0.155
Pulse width	− 0.129	− 0.284 to 0.024	0.096
Side	− 0.058	− 0.230 to 0.114	0.501
Devices	0.149	0.010 to 0.289	0.036
**Multivariate analysis**			R^2^ = 0.1882
Age	-0.5e-5	− 3e-5 to 2e-5	0.727
Gender	0.053	− 0.113 to 0.220	0.520
Duration	0.285	0.122 to 0.448	0.001

CI: confidence interval.

### Severe adverse events

The authors of 10 studies reported 20 “severe” adverse events. One study used the International Conference on Harmonization of Technical Requirements for Registration of Pharmaceuticals for Human Use: Guidance for Good Clinical Practice to define adverse event severity, while two studies from the same author defined an adverse event as severe if the patient was hospitalized^31,32^. Seven studies did not mention the criteria used to define severity. In our review, adverse event severity was determined using the Common Terminology Criteria for Adverse Events (CTCAE). We applied the criteria for those adverse events that the authors identified as "severe" in their studies. Accordingly, of the 20 severe adverse events reported, only 15 were classified as severe based on this classification.

No severe cardiac AEs probably or possibly caused by taVNS were reported. In a randomized, double-blind controlled trial assessing the efficacy and safety of taVNS versus sham stimulation in 39 epilepsy patients, there was one report of palpitation, which was classified as “severe” because the patient was monitored and treated in an intensive care unit after the administration of nitroglycerine, but they appeared unlikely to be caused by the device^31,33^. Further exploration revealed that the patient had experienced comparable events earlier in the past, and she was dismissed from the hospital without complaint 1 day after normal results were noted in echocardiography, duplex sonography of the carotids, 24 h electrocardiography (ECG) monitoring, and thoracal radiography^31^. Cardiac arrhythmias may occur during taVNS due to parasympathetic innervation of the heart caused by the vagus nerve; however, no relevant ECG alterations were observed in any of the subjects enrolled in this study^31^. In a sham-controlled, double-blind, randomized clinical trial of 52 heart failure patients, two (one from the active taVNS group, one from the sham stimulation group) were hospitalized for persistent atrial fibrillation and underwent cardioversion, and three patients (one from active taVNS group, two from sham stimulation group) were hospitalized for heart failure^34^. The authors of this study reported no device-related side effects^34^ (Table 5).

**Table 5:** Adverse events reported as "severe" by the authors versus our classification based on the Common Terminology Criteria for Adverse Events (CTCAE).

Study	Device	Population	Number of SAEs	SAE from Active/Sham	Type	taVNS related	Grade 1,2 (not severe)	Grade 3 (hospitalization)	Grade 4 (life-threatening)	Grade 5 (death)
Evensen 2021	NEMOS, Cerbomed	Depression	1	Active	Anxiety, worsening depression	Probably		1		
Bauer 2016		Epilepsy	1	25 Hz(high level)	Suspected basal cell carcinoma	Probably or possibly		1		
			1	1 Hz(low level)	Sudden death	No				**1**
			1	25 Hz(high level)	Vestibular neuronitis	Probably or possibly	1			
			1	1 Hz(low level)	Palpitation	Probably or possibly	1			
Sabers 2021		Epilepsy	1	Active	Unexpected death	No				**1**
Kreuzer 2014		Tinnitus	1	Active	Fireworks exposure	No		1		
			1	Active	Elective bowel operation	No		1		
Kreuzer 2012	CM02, Cerbomed	Tinnitus	1	Active	Palpitation	No		1		
Hasan 2015		Schizophrenia	1	Active	Hospitalization	No		1		
			1	Active	Appendectomy	No		1		
			1	Sham	Hospitalization	No		1		
Stavrakis 2022	The Parasym, Parasym	Heart failure	1	Active	Heart failure	No		1		
			2	Sham	Heart failure	No		2		
			1	Active	Persistent atrial fibrillation	No			1	
			1	Sham	Persistent atrial fibrillation	No			1	
Peijing 2014	TENS, Suzhou	Epilepsy	1	Active	Severe dizziness	Probably	1			
Aihua 2014	TENS-200, Suzhou	Epilepsy	1	Active	Severe dizziness	Probably	1			
Early 2018	n/a	Chronic Fatigue Syndrome	1	Active	Sensorineural hearing loss	No	1			

SAE: severe adverse events; n/a: non-applicable; Hz: hertz.

Among the 15 severe adverse events identified by us (CTCAE grade ≥ 3), of which none were from healthy subject studies, only two were classified by the authors as "possibly” or “probably" related to taVNS. Therefore, we used the classic Bradford Hill criteria to analyze the relationship between these two severe adverse events and active taVNS. Our analysis shows that the event of anxiety and depression worsening leading to hospitalization could only address the temporality and plausibility criteria, while the suspected basal cell carcinoma addressed only the temporality criteria based on the information provided by the authors. Therefore, our team concluded that there was no causal relationship between the severe adverse events detected in this review and the taVNS (Table 6).

**Table 6:** Severe adverse events classified by the Common Terminology Criteria for Adverse Events reported to be "probably” or “possibly" related to taVNS by the authors’ versus our classification of relatedness based on the Bradford Hill criteria.

Study	Population	Type	Device	Site	Number of sessions	Duration	Intensity	Frequency (Hz)	Pulse (µs)
Evensen 2021	Depression	Anxiety, worsening depression leading to hospitalization	NEMOS, Cerbomed	Left cymba concha	28	Mean: 3.6 (0.9) hours (recommended 4 h)	Adjustable	25	n/a
Bauer 2016	Epilepsy	Suspected basal cell carcinoma			140	4 h	Adjustable	25	0.25

Type	1. Strength	2. Consistency	3. Specificity	4. Temporality	5. Biological gradient	6. Plausibility	7. Coherence	8. Experiment	9. Analogy
Anxiety, worsening depression leading to hospitalization	Weak association since it was only one specific study of a specific population reporting	No consistency	Low specificity, as the case could be caused by different factors	Temporality effects were observed during the administration	No observation of dose–response	No purely biological explanations for the relationship, one possible explanation would be the resistance and fear of the subject in using the device	No biological evidence of the relationship	No experimental evidence of this relationship	No analogy of the relationship was found
**Suspected** basal cell carcinoma	Weak association since it was only one specific study of a specific population reporting	No consistency	Low specificity, as the case could be caused by different factors	There is temporality, since the effects were observed during the administration	No observation of dose–response	No biological plausibility	No biological evidence of the relationship	No experimental evidence of this relationship	No analogy of the relationship was found

Hz: Hertz; µs: micro-seconds.

### Quality assessment

In our review, most of the studies that we assessed the risk of bias were randomized clinical trials (n = 55). The overall evaluation consisted in 13 studies with a low risk of bias, 15 studies with some concerns, and 27 studies with a high risk of bias. The selective reporting (D5) was the domain with less problems detected, while the deviation from the intended interventions (D2), missing outcome data (D3), and outcomes measurement (D4) were the domains with major problems. Most of the studies did not provide enough or clear information on these domains (Supplementary material 5).

## Discussion

We conducted the first systematic review and meta-analysis to evaluate specifically taVNS safety. Our study reviewed 167 articles describing taVNS use in human subjects and aimed to assess its safety and identify associated AEs. To analyze our data at different stages due to the significant discrepancy in reporting techniques, we categorized studies that (1) failed to report the absence or presence of AEs, (2) reported no AEs, and (3) reported at least one AE. Our main finding was that taVNS is a safe technique and does not increase the risk of developing and AE when compared to controls. Considering all the included studies, we detected that no severe adverse events were related to the stimulation according to the CTCAE classification and Bradford Hill’s criteria. We also found that more than half of the studies did not mention the presence or absence of AEs, and these studies did not differ from those that reported no AE or the presence of at least one AE.

### Studies not reporting adverse events

More than half of the studies included in this review did not report any type of assessment of AEs, and it is evident that the inconsistency in AE reporting seen throughout many clinical trials translates into the taVNS field^35^. Interestingly, it has been shown that when studies do not publish AE data, a larger number of adverse events are seen in unpublished versus reported data^36^. In our review, however, evidence points to the opposite trend; trials on taVNS that did not report AE data were, for the most part, performed in healthy populations and undergoing 1–2 sessions. Thus, it is more likely that the taVNS trials not reporting AE data, did not do so because no AEs were experienced. Moreover, when we contacted the authors of trials that did not publish any AE data, the majority of those who replied stated that no AEs were experienced by the subjects in their trials, thus bolstering the assumption that the lack of reporting of AEs is more likely related to a lack of AEs rather than the omission of experienced AEs. Nonetheless, a significant reporting bias and inconsistencies were seen within taVNS studies, suggesting the need for a more standardized format for reporting AEs in this field.

Based on our incidence analysis per cumulative exposure time. The most frequently reported adverse event in the included studies were ear pain, headache, and tingling. Therefore, local AEs were the most frequent type, and most of them were thought to be related to the intervention delivery, specifically to the type of electrodes used for stimulation.

### Safety assessment methods

Many taVNS trials did not specify their safety assessment methods. Among the reported, there were qualitative and quantitative methods. Quantitative scales were based on a patient's numerical rating of common side effects.

Fewer studies mentioned the criteria used to define the AEs severity. A review article published by Redgrave in 2018 defined “serious adverse events'' according to the original authors’ reports. In this review, among the 10 studies that reported “serious adverse events,” only two used the International Conference on Harmonization of Technical Requirements for Registration of Pharmaceuticals for Human Use: Guidance for Good Clinical Practice)^32,37^. One of these studies by Hasan et al. was also included in our analysis, whereas another study using cervical tVNS was excluded. Two studies from the same author defined an adverse event to be “severe” if the patient was hospitalized^31,32^. Otherwise, most studies (7/10) reported at least one “severe AE,” but did not mention their criteria of defining severity. In our review, the severity of the adverse events was determined according to the CTCAE. For future advancement in the field of taVNS to guarantee safety, a consistent definition and practice guideline for AE monitoring must be developed under consensus. Our analysis indicates a low incidence of any type of adverse events in patients exposed to taVNS. The 10 most frequent types were ear pain, headache, tingling, dizziness, skin redness, fatigue, prickling, pressure, itching, and unpleasant feeling. According to our incidence findings, this study is the first to suggest an evidence-based taVNS adverse events questionnaire to actively monitoring patient safety during taVNS. Our questionnaire is based on asking actively the most common types of adverse events accordingly to our review, the patient’s opinion about their relatedness with the treatment, and the classification of severity based on subjects’ opinion guided by the Common Terminology Criteria for Adverse Events (CTCAE). Furthermore, an open question for any additional adverse event experienced by the subject is applied (see supplementary material 6).

### Effects of active taVNS on AE development

Our meta-analysis of 35 studies identified no pooled risk difference between active taVNS and control groups. Only two studies individually showed that taVNS had a small statistically significant effect on AE development. Busch et al. (2012) reported only cases of slight pain, pressure, prickling, itching, trickling, strange feelings, and irritation on swallowing, mostly in both groups. Song et al. detected events such as headache and nausea with only one case each of vomiting, ear pain, dizziness, vertigo, and fatigue, most of which were present in both groups. The two studies did not report severe AEs. When analyzed individually, no specific adverse event subgroup was more likely to appear due to active taVNS. Moreover, regarding the intensity of AE, our pooled analysis showed no differences in AE intensities between active taVNS and controls. Therefore, our analysis conveyed that active taVNS and controls have no differences in the AE development risk and in the AE intensities.

### Factors associated to the development of adverse events

Our multivariate regression analysis revealed a significant association between duration of taVNS stimulation and the number of adverse events experienced per person. This association was not determined by the previous systematic review due to underreporting of tVNS doses^11^. However, considering the additional studies included in our review, more data was able to be gathered regarding duration. Moreover, it is reasonable to assume that the longer the exposure to taVNS, the higher likelihood of a subject developing an adverse event, something that is shown through our analysis. Interestingly, clinically important covariates such as age of participants in the included studies and the prevalence of more female subjects, did not also show a strong or significant relationship with adverse events per person undergoing active taVNS. This result suggests that taVNS can potentially be equally safe for all age ranges, with no difference in gender. It is worth mentioning, however, most studies in our sample included subjects between the ages of 20 and 60 years old, so this suggestion cannot be extrapolated to all age groups.

Regarding transcutaneous cervical VNS, concern may persist about stimulation of the neck. Because transcutaneous vagus nerve stimulation must pass through the skin barrier, relatively strong electrical currents are required, thus nearby non-vagal nerves in the neck may be co-stimulated, possibly including efferent cervical fibers^10^. Similar to the invasive method, transcutaneous cervical VNS stimulates the cervical trunk, thereby activating both efferent and afferent vagus nerve fibers in the carotid sheath^38^. Because it is possible that motor efferents innervating the sinoatrial node are stimulated, there are questions as to whether the cervical approach would be truly safe, and further investigation are needed in this aspect^13^. Because taVNS does not stimulate the neck directly, this approach may have a different safety profile from tcVNS, however its safety had not been reviewed independently until the current study.

Transcutaneous cervical VNS is most delivered via a handheld portable device positioned over the vagus nerve located adjacent to the carotid artery^10^. The most common device (GammaCore®, electroCore, Inc.) is FDA-approved for the treatment of migraine and episodic cluster headache^10^. The device must be held by the patient over the target position, during the entire stimulation. Because the device is self-administered, patients may not correctly stimulate the vagus nerve, by placing the device in the wrong position. Patients also cannot hold a device for long periods of time, repetitively, hence, there is a limitation to the amount of stimulation that can be delivered. In our analysis, 30 studies described only one session of taVNS, and 28 studies applied them to healthy subjects. Noting that most studies aiming to treat certain diseases used multiple stimulation sessions, future devices are more likely to be designed as a convenient take-home device, once proved that the device is safe to use. Therefore, easy and accurate application would become more important when devices are widely applied to patients, and taVNS has advantages in this regard.

According to our analysis, the most common stimulation site was the cymba concha, known as the only site, that is exclusively innervated by the auricular branch of the vagus nerve^12^. Other studies used either commercial or off-label custom devices to stimulate the cavum concha, external auditory meatus, or tragus. These regions are not only supplied by the auricular branch of the vagus nerve, but also by branches of the auriculotemporal nerve, glossopharyngeal nerve, facial nerve, and cervical nerves^39^. We established that regardless of the stimulation site, only non-severe side effects such as local skin problems, headache, and dizziness were observed.

As shown in our analysis, many different electrode forms (ear inserts, ear clips, headphones, etc.) have been introduced for taVNS. According to a previous study on earbud electrodes, given their safety, preliminary efficacy, and comfort results, they warrant further development and investigation. This is because mechanical pinching from electrode clips might cause discomfort, and small stainless steel ball electrodes can cause discomfort due to high current densities at the electrode–skin interface^40^. In our review, there was one case of hand pain related to device application, and minor reports of earclip discomfort. Univariate regression analysis showed that specific taVNS devices were significantly more likely to lead to AEs than off-label devices, but multivariate regression adjusted for age, sex, and duration showed no significant influence. Our review found that taVNS methods up to date are safe and tolerable, leaving room for future developers to improve the reported patient discomforts.

It is generally believed that taVNS must not be administered to the right ear, since efferent vagal fibers on the right side regulate heart rate^41,42^. Previous animal studies reported that right side stimulation more often resulted in bradycardia^38,43^. Therefore, most studies simply avoid the right side for safety concerns; concordantly, most of the studies (71.3%) examined here used the left side. Although few studies used the right or bilateral sides, our analysis revealed that they carry no additional risk. Previous studies reported that stimulation of the right side does not cause severe adverse events^31^. It is worth mentioning that studies applying bilateral cervical tVNS show cardiovascular side effects, which may explain why the preference for unilateral tVNS has been translated to taVNS^44^. However, our analysis and review revealed that bilateral taVNS did not significantly increase the likelihood of cardiovascular events. In fact, no cardiac issues were reported, which has traditionally been a concern for many researchers. This indicates that stimulation of the auricular branch may not directly affect the heart but rather have more afferent fibers that may break this projection unlike the cervical branch of the vagus nerve.

### Severe adverse events

Of the 167 studies reviewed, we detected only two adverse events that we classified as severe in two different studies. Evensen et al. evaluated the safety and feasibility of taVNS in 15 patients with major depression from an inpatient psychiatric center and an outpatient psychiatric clinic. They observed an anxiety attack leading to hospitalization in the active taVNS group that only followed the temporality and partially followed the plausibility of the nine criteria proposed by Bradford Hill^25^. Another severe adverse event was reported by Bauer et al. The authors assessed the efficacy and safety of taVNS in patients with drug-resistant epilepsy. During the stimulation period, they detected in one patient a skin lesion that was suspected to be a basal cell carcinoma. The event only followed the temporality of the 9 criteria and the authors stated that the histology was not confirmed. Our team contacted the authors for further information and histology results with no answers until the submission of this review. Therefore, we conclude that both adverse events cited, besides severe, were not caused by the intervention based on those criteria.

Unlike the traditional concerns that researchers have with taVNS, there have been no reports of severe cardiac AEs that were associated with taVNS. Except for very few patients with underlying heart diseases and recurrent symptoms, we were unable to identify any severe cardiac issues in on this review. Our results confirm previous findings that instantaneous heart rate (HR), and systolic, diastolic, and mean blood pressures are not significantly modified by taVNS, therefore taVNS is safe and tolerable^45^.

### Limitations

As most studies included in our analysis did not distinguish between adverse effect and adverse event, it was difficult to determine the causality of each side effect. Although our attempt to validate casualty of severe adverse events used a scale of relatedness, it is still difficult to determine the true relationship, since only limited information was provided by the original authors. In future studies not employing a sham group, another useful approach would be to correlate AEs with the intervention dosage.

Another major limitation of the literature was the heterogeneity among the included studies. Most studies were excluded from the analysis because they did not report any data regarding safety. The device, electrode type, and stimulation parameters were not specified by some reports. In addition, those studies included had varied assessment and reporting methods. Some studies reported the total frequency, events, or patients with AEs, while others reported each AE type. However, some studies reported no results. Also, some studies may have had a risk of publication or reporting bias. Therefore, although many studies were screened in our initial search, many did not report analyzable data and were excluded from the analysis. Despite these limitations, our findings indicate that AEs related to taVNS were mild and their frequency was low.

One implication of our review is that future taVNS studies should collect more data on AEs. The following three aspects are important:

1. *Safety/toxicity* AEs should be monitored actively, using structured questionnaires in which the rater should ask about each specific AE. In this article, we include a proposed questionnaire for AEs based on our findings (see supplementary material 6) that might be useful as it actively assesses AEs and asks subjects to relate the AE with the effects of taVNS.
2. *Blinding* Reporting AEs is necessary to develop better blinding techniques. In fact, most studies allowed patients to adjust the stimulation intensity under their subjective pain threshold. For example, in studies that used low-intensity stimulation as a sham control, it was possible that patients could discern about their group allocation. Researchers and clinicians should therefore further discuss methods of blinded sham stimulation based on future studies.
3. Precisely identify the duration and the number of sessions that the patients were exposed to develop the AE. Therefore, it will be easier to assess the incidence of the AE due to the cumulative exposure to the intervention.

### Conclusion

Our review of AEs associated with taVNS indicated a selective reporting bias as almost half of the studies did not report the presence or absence of any AEs. We attribute this to the absence of AE on those studies. Furthermore, our meta-analysis conveyed that active taVNS and controls were not different in the risk of developing an AE and in the intensity of the events developed. In general, the incidence of AE was low, being ear pain, headache, and tingling the most frequent ones. No severe adverse event was shown to be caused by taVNS. Thus, we postulate that taVNS is a safe and feasible treatment option. However, while taVNS research moves from bench to bedside, it is essential that future studies explore AEs in an active, systematic, and standardized format.

## Supplementary Information

Supplementary Information 1.

Supplementary Information 2.

Supplementary Information 3.

Supplementary Information 4.

Supplementary Information 5.

Supplementary Information 6.

Supplementary Information 7.

Supplementary Information 8.

## Data Availability

All data generated or analyzed during this study are included in the supplementary material 8 of this article.
