# Supplementary material for: Safety of transcutaneous auricular vagus nerve stimulation (taVNS): a systematic review and meta-analysis
Source: Sci Rep. 2022 Dec 21;12:22055. doi: 10.1038/s41598-022-25864-1 (PMC9772204; doi:10.1038/s41598-022-25864-1)
Supplement: Supplementary file 2 — Supplementary Information 2. [file 41598_2022_25864_MOESM2_ESM.pdf]

Safety of transcutaneous auricular vagus nerve stimulation(taVNS): A systematic review and meta-analysis

Angela Yun Kim1#, Anna Marduy2,3#, Paulo S. de Melo#3,4, Anna Carolyna Gianlorenco3,5, Chi Kyung Kim6, Hyuk Choi7,8, Jae-Jun Song1,8, and Felipe Fregni3

#equally contributed authors

SUPPLEMENTARY MATERIAL 1

Comparison of study characteristics and taVNS parameters between studies not reporting AEs, studies reporting no AEs, and studies reporting at least 1 AE, adjusted after authors' replies.

	Studies not reporting AEs (N= 88)	Studies reporting no AEs (N= 44)	Studies reporting at least 1 AE (N=46)	p-value
Gender (average female %)	50.08	53.00	53.03	0.122
Age (mean±SD)	34.40±14.55	33.24±15.17	37.80±16.10	0.333
Sample size (mean±SD)	35.19±23.04	27.18±26.45	44.86±40.45	0.126
Population				
Healthy	58	28	13	**0.001***
Non-healthy	29	16	33	
Study design				
Parallel	22	14	23	
Crossover	47	20	9	
One arm	12	7	13	
Pilot Study	1	1	0	-
Case Report	3	1	1	
Control Group?				
Yes	62	28	29	0.531
No	25	16	17	
Duration				
0-60 min	63	29	26	0.046
> 60 min	17	12	19	
Number of Sessions				
1-2 Sessions	62	27	14	**<0.001***
Repeated Sessions	24	15	32	
Intensity				
				0.771

Defined	23	12	10	
Adjustable	58	27	32	
Frequency				0.996
1-20 Hz	12	6	6	
20-30 Hz	71	37	37	
Pulse Width				0.276
0-250 μs	44	19	17	
300 μs >	23	19	13	
Stimulation Side				**0.004**
Unilateral	61	38	31	
Bilateral	8	0	10	

*Significant after Bonferroni's correction (p < 0.004)

AE: adverse event; SD: standard deviation; Hz: Hertz; μs: micro-seconds.
