# Supplementary material for: Safety of transcutaneous auricular vagus nerve stimulation (taVNS): a systematic review and meta-analysis
Source: Sci Rep. 2022 Dec 21;12:22055. doi: 10.1038/s41598-022-25864-1 (PMC9772204; doi:10.1038/s41598-022-25864-1)

Safety of transcutaneous auricular vagus nerve stimulation(taVNS): A systematic review and meta-analysis

Angela Yun Kim1#, Anna Marduy2,3#, Paulo S. de Melo#3,4, Anna Carolyna Gianlorenco3,5, Chi Kyung Kim6, Hyuk Choi7,8, Jae-Jun Song1,8, and Felipe Fregni3

#equally contributed authors

SUPPLEMENTARY MATERIAL 2

Forest plot of risk differences between active taVNS and control per adverse event subgroup.

1. Risk differences in ear pain

Study	Experimental Events	Experimental Total	Control Events	Control Total	Weight	Risk Difference MH, Random, 95% CI
Aihua 2014	0	30	0	30	4.0%	0.00 [-0.06; 0.06]
Aranow 2020	0	12	0	6	0.3%	0.00 [-0.22; 0.22]
Badran1 2018	0	15	0	15	1.1%	0.00 [-0.12; 0.12]
Badran2 2018	0	20	0	20	1.8%	0.00 [-0.09; 0.09]
Bauer 2016	6	37	3	39	0.7%	0.09 [-0.06; 0.23]
Busch 2012	2	48	1	48	3.3%	0.02 [-0.05; 0.09]
Cao 2021	0	24	0	24	2.6%	0.00 [-0.08; 0.08]
Capone 2014	0	10	0	10	0.5%	0.00 [-0.17; 0.17]
Capone 2017	0	7	0	5	0.2%	0.00 [-0.28; 0.28]
Chang 2021	0	18	0	18	1.5%	0.00 [-0.10; 0.10]
FRØKJÆR 2016	0	18	0	18	1.5%	0.00 [-0.10; 0.10]
Geng 2022	0	14	0	14	1.0%	0.00 [-0.13; 0.13]
Hein1 2013	0	11	0	11	0.6%	0.00 [-0.16; 0.16]
Hein2 2013	0	7	0	8	0.3%	0.00 [-0.22; 0.22]
Huang 2014	0	36	0	36	5.7%	0.00 [-0.05; 0.05]
Jacobs 2015	0	30	0	30	4.0%	0.00 [-0.06; 0.06]
Jiao 2020	2	36	1	36	1.9%	0.03 [-0.06; 0.12]
Jongkees 2018	0	40	0	40	7.0%	0.00 [-0.05; 0.05]
Kutlu 2020	0	24	0	35	3.5%	0.00 [-0.07; 0.07]
Li 2022	0	30	0	30	4.0%	0.00 [-0.06; 0.06]
Pihlaja 2020	0	25	0	25	2.8%	0.00 [-0.07; 0.07]
Ricci 2020	0	8	0	8	0.4%	0.00 [-0.21; 0.21]
Rong 2016	0	69	0	69	20.2%	0.00 [-0.03; 0.03]
Sclocco 2020	0	30	0	30	4.0%	0.00 [-0.06; 0.06]
Sellaro 2015	0	20	0	20	1.8%	0.00 [-0.09; 0.09]
Sellaro 2017	0	24	0	24	2.6%	0.00 [-0.08; 0.08]
Song 2018	3	27	1	25	0.8%	0.07 [-0.07; 0.21]
Stavrakis 2022	0	26	0	26	3.0%	0.00 [-0.07; 0.07]
Steenbergen 2015	0	15	0	15	1.1%	0.00 [-0.12; 0.12]
Sun 2021	0	46	0	46	9.1%	0.00 [-0.04; 0.04]
Thakkar 2020	0	14	0	23	1.4%	0.00 [-0.11; 0.11]
Tran 2018	0	24	0	24	2.6%	0.00 [-0.08; 0.08]
Warren 2019	0	24	0	24	2.6%	0.00 [-0.08; 0.08]
Wu 2020	0	10	0	11	0.6%	0.00 [-0.17; 0.17]
Zhu 2021	0	18	0	18	1.5%	0.00 [-0.10; 0.10]
**Total (95% CI)**		**847**		**861**	**100.0%**	**0.00 [-0.01; 0.01]**

Heterogeneity: $Tau^2 = 0$; $Chi^2 = 2.85$, df = 34 ($P = 1.00$); $I^2 = 0\%$

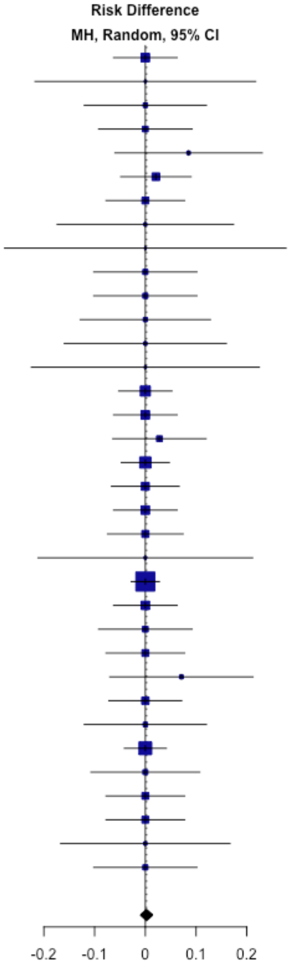

2. Risk differences in dizziness

	Experimental		Control			Risk Difference
**Study**	**Events**	**Total**	**Events**	**Total**	**Weight**	**MH, Random, 95% CI**
Aihua 2014	1	30	0	30	1.9%	0.03 [-0.05; 0.12]
Aranow 2020	0	12	0	6	0.3%	0.00 [-0.22; 0.22]
Badran1 2018	0	15	0	15	1.0%	0.00 [-0.12; 0.12]
Badran2 2018	0	20	0	20	1.8%	0.00 [-0.09; 0.09]
Bauer 2016	5	37	2	39	0.9%	0.08 [-0.05; 0.21]
Busch 2012	0	48	0	48	9.4%	0.00 [-0.04; 0.04]
Cao 2021	0	24	0	24	2.5%	0.00 [-0.08; 0.08]
Capone 2014	0	10	0	10	0.5%	0.00 [-0.17; 0.17]
Capone 2017	0	7	0	5	0.2%	0.00 [-0.28; 0.28]
Chang 2021	0	18	0	18	1.4%	0.00 [-0.10; 0.10]
FRØKJÆR 2016	0	18	0	18	1.4%	0.00 [-0.10; 0.10]
Geng 2022	0	14	0	14	0.9%	0.00 [-0.13; 0.13]
Hein1 2013	0	11	0	11	0.6%	0.00 [-0.16; 0.16]
Hein2 2013	0	7	0	8	0.3%	0.00 [-0.22; 0.22]
Huang 2014	2	36	0	36	1.9%	0.06 [-0.03; 0.14]
Jacobs 2015	0	30	0	30	3.8%	0.00 [-0.06; 0.06]
Jiao 2020	0	36	0	36	5.4%	0.00 [-0.05; 0.05]
Jongkees 2018	0	40	0	40	6.6%	0.00 [-0.05; 0.05]
Kutlu 2020	0	24	0	35	3.3%	0.00 [-0.07; 0.07]
Li 2022	0	30	0	30	3.8%	0.00 [-0.06; 0.06]
Pihlaja 2020	0	25	0	25	2.7%	0.00 [-0.07; 0.07]
Ricci 2020	0	8	0	8	0.3%	0.00 [-0.21; 0.21]
Rong 2016	0	69	0	69	19.2%	0.00 [-0.03; 0.03]
Sclocco 2020	0	30	0	30	3.8%	0.00 [-0.06; 0.06]
Sellaro 2015	0	20	0	20	1.8%	0.00 [-0.09; 0.09]
Sellaro 2017	0	24	0	24	2.5%	0.00 [-0.08; 0.08]
Song 2018	2	27	1	25	1.0%	0.03 [-0.09; 0.16]
Stavrakis 2022	0	26	0	26	2.9%	0.00 [-0.07; 0.07]
Steenbergen 2015	0	15	0	15	1.0%	0.00 [-0.12; 0.12]
Sun 2021	0	46	0	46	8.7%	0.00 [-0.04; 0.04]
Thakkar 2020	0	14	0	23	1.3%	0.00 [-0.11; 0.11]
Tran 2018	0	24	0	24	2.5%	0.00 [-0.08; 0.08]
Warren 2019	0	24	0	24	2.5%	0.00 [-0.08; 0.08]
Wu 2020	0	10	0	11	0.5%	0.00 [-0.17; 0.17]
Zhu 2021	0	18	0	18	1.4%	0.00 [-0.10; 0.10]
**Total (95% CI)**		**847**		**861**	**100.0%**	**0.00 [-0.01; 0.01]**

Heterogeneity: $Tau^2 = 0$; $Chi^2 = 3.74$, df = 34 (P = 1.00); $I^2 = 0\%$

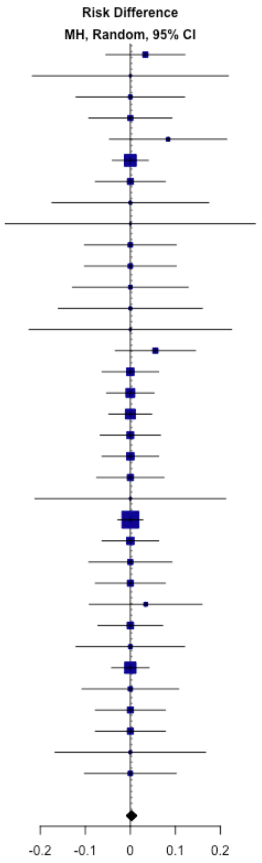

3. Risk differences in skin redness

Study	Experimental Events	Experimental Total	Control Events	Control Total	Weight	Risk Difference MH, Random, 95% CI
Aihua 2014	0	30	0	30	3.6%	0.00 [-0.06; 0.06]
Aranow 2020	0	12	0	6	0.3%	0.00 [-0.22; 0.22]
Badran1 2018	0	15	0	15	1.0%	0.00 [-0.12; 0.12]
Badran2 2018	0	20	0	20	1.7%	0.00 [-0.09; 0.09]
Bauer 2016	3	37	1	39	1.4%	0.06 [-0.05; 0.16]
Busch 2012	0	48	0	48	9.0%	0.00 [-0.04; 0.04]
Cao 2021	0	24	0	24	2.4%	0.00 [-0.08; 0.08]
Capone 2014	0	10	0	10	0.5%	0.00 [-0.17; 0.17]
Capone 2017	0	7	0	5	0.2%	0.00 [-0.28; 0.28]
Chang 2021	0	18	0	18	1.4%	0.00 [-0.10; 0.10]
FRØKJÆR 2016	0	18	0	18	1.4%	0.00 [-0.10; 0.10]
Geng 2022	0	14	0	14	0.9%	0.00 [-0.13; 0.13]
Hein1 2013	0	11	0	11	0.6%	0.00 [-0.16; 0.16]
Hein2 2013	0	7	0	8	0.3%	0.00 [-0.22; 0.22]
Huang 2014	0	36	0	36	5.1%	0.00 [-0.05; 0.05]
Jacobs 2015	0	30	0	30	3.6%	0.00 [-0.06; 0.06]
Jiao 2020	0	36	0	36	5.1%	0.00 [-0.05; 0.05]
Jongkees 2018	0	40	0	40	6.3%	0.00 [-0.05; 0.05]
Kutlu 2020	0	24	0	35	3.2%	0.00 [-0.07; 0.07]
Li 2022	2	30	0	30	1.3%	0.07 [-0.04; 0.17]
Pihlaja 2020	0	25	0	25	2.5%	0.00 [-0.07; 0.07]
Ricci 2020	0	8	0	8	0.3%	0.00 [-0.21; 0.21]
Rong 2016	0	69	0	69	18.2%	0.00 [-0.03; 0.03]
Sclocco 2020	0	30	0	30	3.6%	0.00 [-0.06; 0.06]
Sellaro 2015	0	20	0	20	1.7%	0.00 [-0.09; 0.09]
Sellaro 2017	0	24	0	24	2.4%	0.00 [-0.08; 0.08]
Song 2018	0	27	0	25	2.7%	0.00 [-0.07; 0.07]
Stavrakis 2022	0	26	0	26	2.7%	0.00 [-0.07; 0.07]
Steenbergen 2015	0	15	0	15	1.0%	0.00 [-0.12; 0.12]
Sun 2021	0	46	0	46	8.3%	0.00 [-0.04; 0.04]
Thakkar 2020	0	14	0	23	1.2%	0.00 [-0.11; 0.11]
Tran 2018	0	24	0	24	2.4%	0.00 [-0.08; 0.08]
Warren 2019	0	24	0	24	2.4%	0.00 [-0.08; 0.08]
Wu 2020	1	10	0	11	0.3%	0.10 [-0.13; 0.33]
Zhu 2021	0	18	0	18	1.4%	0.00 [-0.10; 0.10]
**Total (95% CI)**		**847**		**861**	**100.0%**	**0.00 [-0.01; 0.01]**

Heterogeneity: $Tau^2 = 0$; $Chi^2 = 3.31$, df = 34 ($P = 1.00$); $I^2 = 0\%$

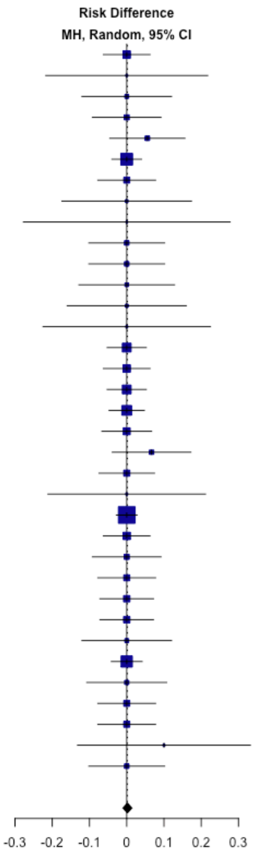

4. Risk differences in headache

Study	Experimental Events	Experimental Total	Control Events	Control Total	Weight	Risk Difference MH, Random, 95% CI
Aihua 2014	0	30	0	30	3.7%	0.00 [-0.06; 0.06]
Aranow 2020	0	12	0	6	0.3%	0.00 [-0.22; 0.22]
Badran1 2018	0	15	0	15	1.0%	0.00 [-0.12; 0.12]
Badran2 2018	0	20	0	20	1.7%	0.00 [-0.09; 0.09]
Bauer 2016	12	37	14	39	0.3%	-0.03 [-0.25; 0.18]
Busch 2012	0	48	0	48	9.2%	0.00 [-0.04; 0.04]
Cao 2021	0	24	0	24	2.4%	0.00 [-0.08; 0.08]
Capone 2014	0	10	0	10	0.5%	0.00 [-0.17; 0.17]
Capone 2017	0	7	0	5	0.2%	0.00 [-0.28; 0.28]
Chang 2021	0	18	0	18	1.4%	0.00 [-0.10; 0.10]
FRØKJÆR 2016	0	18	0	18	1.4%	0.00 [-0.10; 0.10]
Geng 2022	0	14	0	14	0.9%	0.00 [-0.13; 0.13]
Hein1 2013	0	11	0	11	0.6%	0.00 [-0.16; 0.16]
Hein2 2013	0	7	0	8	0.3%	0.00 [-0.22; 0.22]
Huang 2014	0	36	0	36	5.3%	0.00 [-0.05; 0.05]
Jacobs 2015	0	30	0	30	3.7%	0.00 [-0.06; 0.06]
Jiao 2020	1	36	0	36	2.7%	0.03 [-0.05; 0.10]
Jongkees 2018	0	40	0	40	6.5%	0.00 [-0.05; 0.05]
Kutlu 2020	0	24	0	35	3.3%	0.00 [-0.07; 0.07]
Li 2022	0	30	0	30	3.7%	0.00 [-0.06; 0.06]
Pihlaja 2020	0	25	0	25	2.6%	0.00 [-0.07; 0.07]
Ricci 2020	0	8	0	8	0.3%	0.00 [-0.21; 0.21]
Rong 2016	0	69	0	69	18.8%	0.00 [-0.03; 0.03]
Sclocco 2020	0	30	0	30	3.7%	0.00 [-0.06; 0.06]
Sellaro 2015	0	20	0	20	1.7%	0.00 [-0.09; 0.09]
Sellaro 2017	0	24	0	24	2.4%	0.00 [-0.08; 0.08]
Song 2018	3	27	1	25	0.7%	0.07 [-0.07; 0.21]
Stavrakis 2022	0	26	0	26	2.8%	0.00 [-0.07; 0.07]
Steenbergen 2015	0	15	0	15	1.0%	0.00 [-0.12; 0.12]
Sun 2021	0	46	0	46	8.5%	0.00 [-0.04; 0.04]
Thakkar 2020	0	14	0	23	1.3%	0.00 [-0.11; 0.11]
Tran 2018	0	24	0	24	2.4%	0.00 [-0.08; 0.08]
Warren 2019	0	24	0	24	2.4%	0.00 [-0.08; 0.08]
Wu 2020	0	10	0	11	0.5%	0.00 [-0.17; 0.17]
Zhu 2021	0	18	0	18	1.4%	0.00 [-0.10; 0.10]
**Total (95% CI)**		**847**		**861**	**100.0%**	**0.00 [-0.01; 0.01]**

Heterogeneity: $Tau^2 = 0$; $Chi^2 = 1.59$, df = 34 (P = 1.00); $I^2 = 0\%$

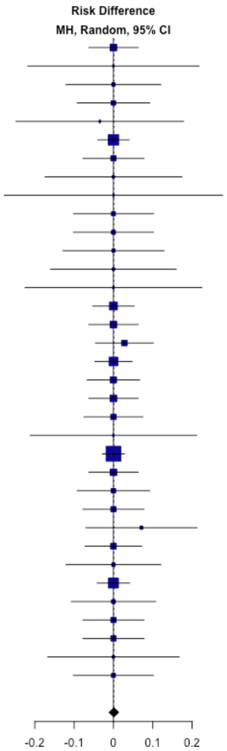

Supplement: Supplementary file 3 — Supplementary Information 3. [file 41598_2022_25864_MOESM3_ESM.pdf]
