# Supplementary material for: Safety of transcutaneous auricular vagus nerve stimulation (taVNS): a systematic review and meta-analysis
Source: Sci Rep. 2022 Dec 21;12:22055. doi: 10.1038/s41598-022-25864-1 (PMC9772204; doi:10.1038/s41598-022-25864-1)

Safety of transcutaneous auricular vagus nerve stimulation(taVNS): A systematic review and meta-analysis

Angela Yun Kim1#, Anna Marduy2,3#, Paulo S. de Melo#3,4, Anna Carolyna Gianlorenco3,5, Chi Kyung Kim6, Hyuk Choi7,8, Jae-Jun Song1,8, and Felipe Fregni3

#equally contributed authors

SUPPLEMENTARY MATERIAL 3

Forest plot of standard mean differences of intensities of adverse events between active taVNS and control per adverse event subgroup.

1. Standard mean differences of headache intensity

	Experimental			Control				Std. Mean Difference
**Study**	**Mean**	**SD**	**Total**	**Mean**	**SD**	**Total**	**Weight**	**IV, Random, 95% CI**
Burger 2018	.	.	42	.	.	43	0.0%	
D'Agostini 2021b	0.88	1.3900	66	0.73	1.2900	66	16.4%	0.11 [-0.23; 0.45]
D'Agostini 2021a	2.09	1.8000	32	1.97	1.7600	35	12.8%	0.07 [-0.41; 0.55]
Fischer 2018	1.50	0.2200	21	1.81	0.2500	21	9.0%	-1.29 [-1.96; -0.62]
Giraudier 2020	1.83	1.4200	30	2.03	1.3800	30	12.2%	-0.14 [-0.65; 0.37]
Konjusha 2022	1.37	0.1000	45	1.40	0.1000	45	14.4%	-0.30 [-0.71; 0.12]
Maraver 2020	1.72	1.0300	43	1.70	0.9400	43	14.2%	0.02 [-0.40; 0.44]
Sharon 2021	1.82	0.2000	24	1.89	0.2200	24	10.8%	-0.33 [-0.90; 0.24]
Ventura-Bort 2018	1.50	1.5000	21	1.85	1.1800	21	10.1%	-0.25 [-0.86; 0.35]
**Total (95% CI)**			**324**			**328**	**100.0%**	**-0.21 [-0.46; 0.05]**

Heterogeneity: Tau^2 = 0.0741; Chi^2 = 15.77, df = 7 (P = 0.03); I^2 = 56%

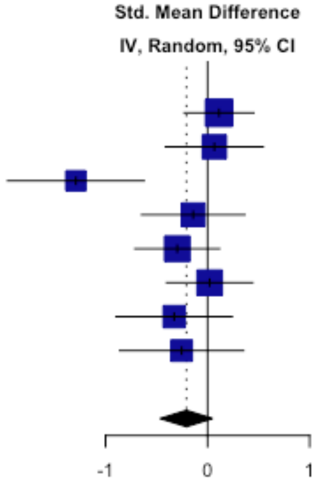

2. Standard mean differences of dizziness intensity

	Experimental			Control				Std. Mean Difference
**Study**	**Mean**	**SD**	**Total**	**Mean**	**SD**	**Total**	**Weight**	**IV, Random, 95% CI**
Burger 2018	.	.	42	.	.	43	0.0%	
D'Agostini 2021b	.	.	66	.	.	66	0.0%	
D'Agostini 2021a	.	.	32	.	.	35	0.0%	
Fischer 2018	1.71	0.2100	21	1.67	0.2400	21	22.9%	0.17 [-0.43; 0.78]
Giraudier 2020	1.67	1.1500	30	2.27	1.5500	30	29.6%	-0.43 [-0.95; 0.08]
Konjusha 2022	.	.	45	.	.	45	0.0%	
Maraver 2020	.	.	43	.	.	43	0.0%	
Sharon 2021	1.82	0.2200	24	1.97	0.2500	24	24.5%	-0.63 [-1.21; -0.05]
Ventura-Bort 2018	1.55	0.6000	21	1.70	1.1300	21	23.0%	-0.16 [-0.77; 0.44]
**Total (95% CI)**			**324**			**328**	**100.0%**	**-0.28 [-0.61; 0.05]**

Heterogeneity: Tau^2 = 0.0262; Chi^2 = 4.01, df = 3 (P = 0.26); I^2 = 25%

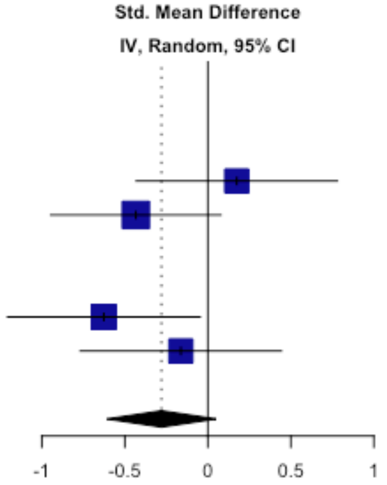

3. Standard mean differences of skin irritation intensity

Study	Experimental Mean	Experimental SD	Experimental Total	Control Mean	Control SD	Control Total	Weight	Std. Mean Difference IV, Random, 95% CI
Burger 2018	.	.	42	.	.	43	0.0%	
D'Agostini 2021b	.	.	66	.	.	66	0.0%	
D'Agostini 2021a	.	.	32	.	.	35	0.0%	
Fischer 2018	2.00	0.3000	21	1.30	0.2100	21	23.8%	2.65 [1.80; 3.50]
Giraudier 2020	1.77	1.4500	30	1.93	1.5500	30	25.7%	-0.11 [-0.61; 0.40]
Konjusha 2022	.	.	45	.	.	45	0.0%	
Maraver 2020	.	.	43	.	.	43	0.0%	
Sharon 2021	2.17	0.2800	24	2.15	0.2700	24	25.4%	0.07 [-0.49; 0.64]
Ventura-Bort 2018	1.80	1.0000	21	1.15	0.4900	21	25.1%	0.81 [0.18; 1.44]
**Total (95% CI)**			**324**			**328**	**100.0%**	**0.82 [-0.38; 2.03]**

Heterogeneity: $Tau^2 = 1.3985$; $Chi^2 = 33.36$, df = 3 ($P < 0.01$); $I^2 = 91\%$

Std. Mean Difference IV, Random, 95% CI

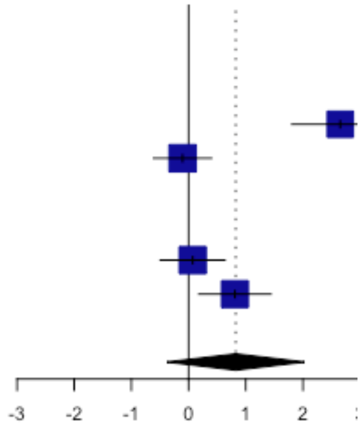

4. Standard mean differences of nausea intensity

Study	Experimental Mean	Experimental SD	Experimental Total	Control Mean	Control SD	Control Total	Weight	Std. Mean Difference IV, Random, 95% CI
Burger 2018	.	.	42	.	.	43	0.0%	
D'Agostini 2021b	0.38	1.0200	66	0.28	0.7900	66	17.9%	0.11 [-0.23; 0.45]
D'Agostini 2021a	1.53	1.3200	32	1.09	0.5100	35	12.4%	0.44 [-0.04; 0.93]
Fischer 2018	1.19	0.1300	21	1.23	0.1200	21	9.3%	-0.31 [-0.92; 0.30]
Giraudier 2020	1.17	0.7500	30	1.30	0.7500	30	11.8%	-0.17 [-0.68; 0.34]
Konjusha 2022	1.06	0.0300	45	1.04	0.0300	45	14.5%	0.66 [0.24; 1.09]
Maraver 2020	1.25	0.7200	43	1.25	0.6200	43	14.6%	0.00 [-0.42; 0.42]
Sharon 2021	1.67	0.1900	24	1.63	0.1900	24	10.2%	0.21 [-0.36; 0.77]
Ventura-Bort 2018	1.20	0.6100	21	1.23	0.5500	21	9.3%	-0.05 [-0.66; 0.55]
**Total (95% CI)**			**324**			**328**	**100.0%**	**0.14 [-0.08; 0.36]**

Heterogeneity: $Tau^2 = 0.0408$; $Chi^2 = 11.72$, df = 7 ($P = 0.11$); $I^2 = 40\%$

Std. Mean Difference IV, Random, 95% CI

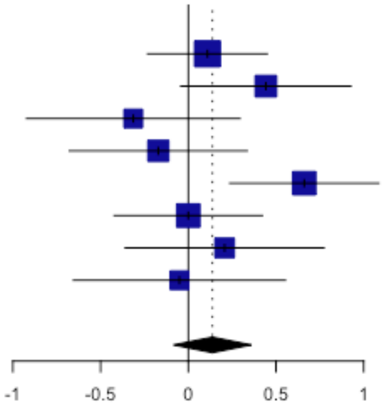

5. Standard mean differences of stinging sensation intensity

	Experimental			Control				Std. Mean Difference
Study	Mean	SD	Total	Mean	SD	Total	Weight	IV, Random, 95% CI
Burger 2018	.	.	42	.	.	43	0.0%	
D'Agostini 2021b	.	.	66	.	.	66	0.0%	
D'Agostini 2021a	.	.	32	.	.	35	0.0%	
Fischer 2018	3.00	0.4700	21	2.05	1.4000	21	16.2%	0.89 [0.26; 1.53]
Giraudier 2020	2.87	1.9400	30	2.80	1.7300	30	16.9%	0.04 [-0.47; 0.54]
Konjusha 2022	2.15	0.1800	45	1.75	0.1500	45	16.7%	2.39 [1.85; 2.94]
Maraver 2020	2.62	1.3800	43	2.65	1.4900	43	17.3%	-0.02 [-0.44; 0.40]
Sharon 2021	2.22	0.2200	24	2.18	0.2300	24	16.6%	0.17 [-0.39; 0.74]
Ventura-Bort 2018	2.87	2.0700	21	1.95	1.3900	21	16.3%	0.51 [-0.10; 1.13]
**Total (95% CI)**			**324**			**328**	**100.0%**	**0.66 [-0.08; 1.39]**

Heterogeneity: $Tau^2 = 0.7654$; $Chi^2 = 57.46$, df = 5 ($P < 0.01$); $I^2 = 91\%$

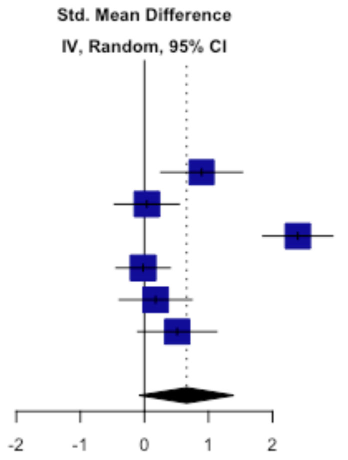

6. Standard mean differences of neck pain intensity

	Experimental			Control				Std. Mean Difference
Study	Mean	SD	Total	Mean	SD	Total	Weight	IV, Random, 95% CI
Burger 2018	.	.	42	.	.	43	0.0%	
D'Agostini 2021b	0.72	1.3000	66	0.88	1.2000	66	15.3%	-0.13 [-0.47; 0.21]
D'Agostini 2021a	2.63	1.8400	32	1.80	1.5100	35	14.5%	0.49 [0.00; 0.98]
Fischer 2018	1.47	0.2000	21	1.30	0.1100	21	13.5%	1.03 [0.39; 1.68]
Giraudier 2020	1.80	1.4900	30	1.30	0.7500	30	14.4%	0.42 [-0.09; 0.93]
Konjusha 2022	1.26	0.0800	45	1.37	0.0900	45	14.7%	-1.28 [-1.74; -0.83]
Maraver 2020	.	.	43	.	.	43	0.0%	
Sharon 2021	1.87	0.1600	24	1.97	0.1900	24	13.9%	-0.56 [-1.14; 0.02]
Ventura-Bort 2018	1.50	0.2000	21	1.30	0.4700	21	13.7%	0.54 [-0.07; 1.16]
**Total (95% CI)**			**324**			**328**	**100.0%**	**0.06 [-0.52; 0.64]**

Heterogeneity: $Tau^2 = 0.5433$; $Chi^2 = 53.50$, df = 6 ($P < 0.01$); $I^2 = 89\%$

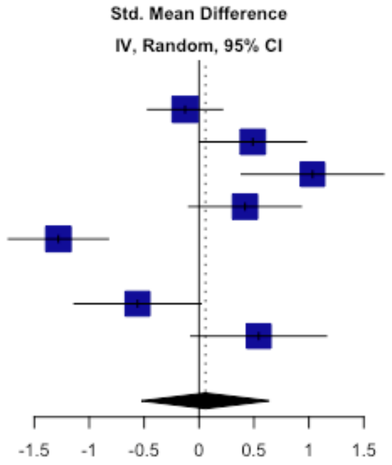

7. Standard mean differences of unpleasant feeling intensity

	Experimental			Control				Std. Mean Difference
Study	Mean	SD	Total	Mean	SD	Total	Weight	IV, Random, 95% CI
Burger 2018	.	.	42	.	.	43	0.0%	
D'Agostini 2021b	1.80	1.6700	66	1.63	1.4900	66	16.8%	0.11 [-0.23; 0.45]
D'Agostini 2021a	2.91	1.6100	32	2.06	1.3700	35	12.5%	0.56 [0.07; 1.05]
Fischer 2018	2.15	1.4200	21	1.81	0.2700	21	9.8%	0.33 [-0.28; 0.94]
Giraudier 2020	1.67	1.1500	30	2.23	1.4500	30	11.9%	-0.42 [-0.93; 0.09]
Konjusha 2022	1.60	0.1000	45	1.60	0.1300	45	14.6%	0.00 [-0.41; 0.41]
Maraver 2020	2.21	1.1600	43	2.09	1.2500	43	14.3%	0.10 [-0.32; 0.52]
Sharon 2021	2.52	0.2900	24	2.71	0.3100	24	10.4%	-0.62 [-1.20; -0.04]
Ventura-Bort 2018	1.94	1.1300	21	1.65	1.0400	21	9.8%	0.26 [-0.35; 0.87]
**Total (95% CI)**			**324**			**328**	**100.0%**	**0.05 [-0.20; 0.29]**

Heterogeneity: $Tau^2 = 0.0619$; $Chi^2 = 14.14$, df = 7 ($P = 0.05$); $I^2 = 50\%$

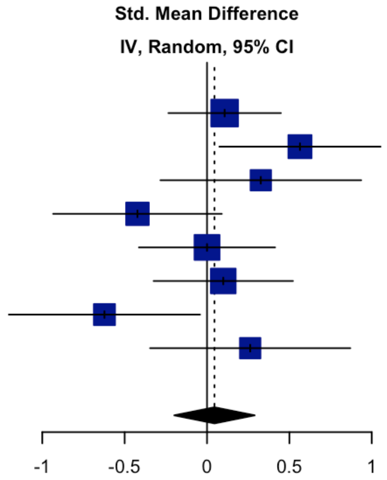

8. Standard mean differences of fluctuation feeling intensity

Study	Experimental Mean	Experimental SD	Experimental Total	Control Mean	Control SD	Control Total	Weight	Std. Mean Difference IV, Random, 95% CI
Burger 2018	.	.	42	.	.	43	0.0%	
D'Agostini 2021b	.	.	66	.	.	66	0.0%	
D'Agostini 2021a	.	.	32	.	.	35	0.0%	
Fischer 2018	1.85	0.2700	21	1.54	0.2400	21	31.8%	1.19 [0.53; 1.85]
Giraudier 2020	1.53	1.0100	30	1.73	1.3400	30	35.2%	-0.17 [-0.67; 0.34]
Konjusha 2022	.	.	45	.	.	45	0.0%	
Maraver 2020	.	.	43	.	.	43	0.0%	
Sharon 2021	.	.	24	.	.	24	0.0%	
Ventura-Bort 2018	1.65	0.8800	21	1.37	0.8100	21	33.0%	0.32 [-0.28; 0.93]
**Total (95% CI)**			**324**			**328**	**100.0%**	**0.43 [-0.35; 1.20]**

Heterogeneity: $Tau^2 = 0.3760$; $Chi^2 = 10.19$, df = 2 ($P < 0.01$); $I^2 = 80\%$

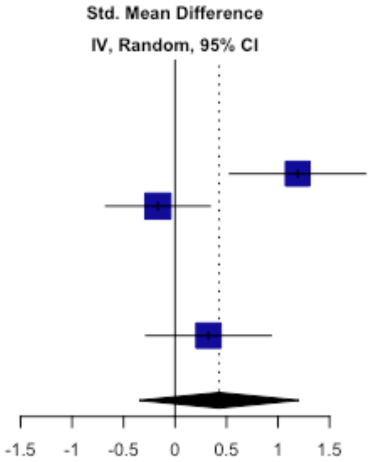

9. Standard mean differences of burning sensation intensity

Study	Experimental Mean	Experimental SD	Experimental Total	Control Mean	Control SD	Control Total	Weight	Std. Mean Difference IV, Random, 95% CI
Burger 2018	.	.	42	.	.	43	0.0%	
D'Agostini 2021b	0.91	1.4300	66	1.00	1.4700	66	25.5%	-0.06 [-0.40; 0.28]
D'Agostini 2021a	3.19	1.9100	32	1.69	1.1600	35	24.8%	0.95 [0.44; 1.46]
Fischer 2018	.	.	21	.	.	21	0.0%	
Giraudier 2020	.	.	30	.	.	30	0.0%	
Konjusha 2022	1.86	0.1400	45	1.53	0.1200	45	24.5%	2.51 [1.95; 3.07]
Maraver 2020	2.25	1.1900	43	1.79	1.1400	43	25.2%	0.39 [-0.04; 0.82]
Sharon 2021	.	.	24	.	.	24	0.0%	
Ventura-Bort 2018	.	.	21	.	.	21	0.0%	
**Total (95% CI)**			**324**			**328**	**100.0%**	**0.93 [-0.16; 2.02]**

Heterogeneity: $Tau^2 = 1.1833$; $Chi^2 = 62.13$, df = 3 ($P < 0.01$); $I^2 = 95\%$

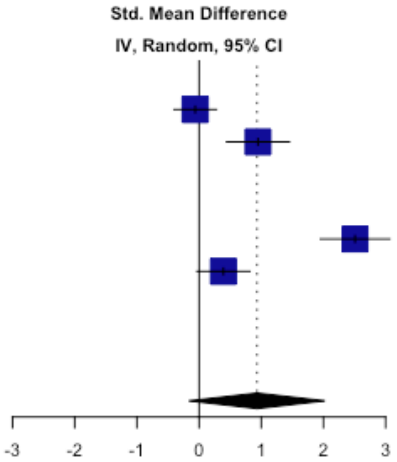

10. Standard mean differences of muscle contraction intensity

Study	Experimental Mean	Experimental SD	Experimental Total	Control Mean	Control SD	Control Total	Weight	Std. Mean Difference IV, Random, 95% CI
Burger 2018	.	.	42	.	.	43	0.0%	
D'Agostini 2021b	.	.	66	.	.	66	0.0%	
D'Agostini 2021a	.	.	32	.	.	35	0.0%	
Fischer 2018	1.62	0.2000	21	1.70	0.2100	21	12.3%	-0.38 [-0.99; 0.23]
Giraudier 2020	2.27	0.2000	30	1.93	1.4600	30	16.6%	0.32 [-0.19; 0.83]
Konjusha 2022	1.28	0.0800	45	1.28	0.0800	45	23.0%	0.00 [-0.41; 0.41]
Maraver 2020	1.83	0.9700	43	1.51	0.8000	43	22.0%	0.36 [-0.07; 0.78]
Sharon 2021	2.10	0.2000	24	2.00	0.2100	24	13.6%	0.48 [-0.09; 1.05]
Ventura-Bort 2018	1.60	0.9500	21	1.70	0.9300	21	12.5%	-0.10 [-0.71; 0.50]
**Total (95% CI)**			**324**			**328**	**100.0%**	**0.14 [-0.09; 0.37]**

Heterogeneity: $Tau^2 = 0.0161$; $Chi^2 = 6.71$, df = 5 (P = 0.24); $I^2 = 25\%$

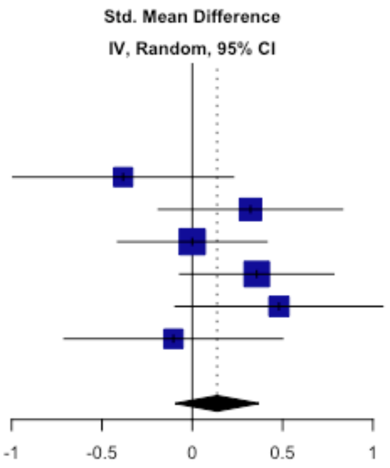

11. Standard mean differences of changes in concentration intensity

Study	Experimental Mean	Experimental SD	Experimental Total	Control Mean	Control SD	Control Total	Weight	Std. Mean Difference IV, Random, 95% CI
Burger 2018	.	.	42	.	.	43	0.0%	
D'Agostini 2021b	29.12	25.7300	66	28.66	23.2700	66	18.7%	0.02 [-0.32; 0.36]
D'Agostini 2021a	21.84	27.1000	32	6.34	16.0000	35	17.1%	0.70 [0.20; 1.19]
Fischer 2018	3.71	0.4000	21	3.52	0.3700	21	15.7%	0.48 [-0.13; 1.10]
Giraudier 2020	1.93	1.5500	30	3.47	1.6800	30	16.6%	-0.94 [-1.48; -0.41]
Konjusha 2022	.	.	45	.	.	45	0.0%	
Maraver 2020	.	.	43	.	.	43	0.0%	
Sharon 2021	4.02	0.3900	24	3.73	0.3600	24	16.0%	0.76 [0.17; 1.35]
Ventura-Bort 2018	3.55	1.7300	21	3.45	1.7000	21	15.8%	0.06 [-0.55; 0.66]
**Total (95% CI)**			**324**			**328**	**100.0%**	**0.17 [-0.33; 0.68]**

Heterogeneity: $Tau^2 = 0.3205$; $Chi^2 = 26.56$, df = 5 (P < 0.01); $I^2 = 81\%$

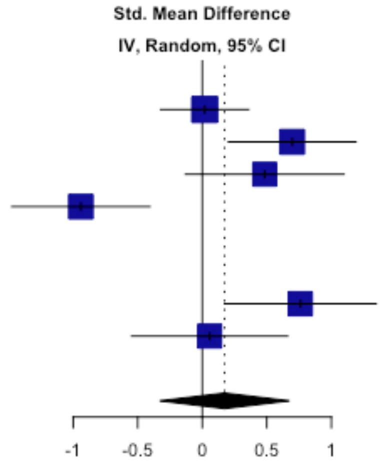

Supplement: Supplementary file 4 — Supplementary Information 4. [file 41598_2022_25864_MOESM4_ESM.pdf]
