# Supplementary material for: Safety of transcutaneous auricular vagus nerve stimulation (taVNS): a systematic review and meta-analysis
Source: Sci Rep. 2022 Dec 21;12:22055. doi: 10.1038/s41598-022-25864-1 (PMC9772204; doi:10.1038/s41598-022-25864-1)
Supplement: Supplementary file 5 — Supplementary Information 5. [file 41598_2022_25864_MOESM5_ESM.pdf]

Safety of transcutaneous auricular vagus nerve stimulation(taVNS): A systematic review and meta-analysis

Angela Yun Kim1#, Anna Marduy2,3#, Paulo S. de Melo#3,4, Anna Carolyna Gianlorenco3,5, Chi Kyung Kim6, Hyuk Choi7,8, Jae-Jun Song1,8, and Felipe Fregni3

#equally contributed authors

SUPPLEMENTARY MATERIAL 4

Output Risk of Bias Tool 2.0 figure.

Study ID	D1	D2	D3	D4	D5	Overall
Aihua et al 2014	!	!	!	+	-	-
Aranow et al 2020	+	!	+	+	+	!
Badran et al 2018	!	+	+	-	+	-
Bandran et al 2020	-	!	+	!	+	-
Barbella et al 2018	!	!	+	!	+	!
Bauer et al 2016	+	+	!	+	+	+
Burger et al 2016	+	!	+	+	+	!
Burger et al 2018	+	!	+	!	+	!
Burger et al 2019	+	!	+	!	+	!
Busch et al 2012	+	+	+	+	-	-
Cao et al 2021	+	+	+	!	+	!
Capone et al 2014	!	-	-	+	!	-
Capone et al 2017	!	!	+	+	+	!
Chang et al 2021	!	-	-	-	!	-
Colzoato et al 2018	!	-	-	-	!	-
D'Agostini et al 2021	!	-	+	-	!	-
D'Agostini et al 2022	-	+	!	+	+	-
Dalgleish et al 2021	!	-	-	-	!	-
Fischer et al 2018	!	-	-	-	+	-
FRØKJÆR et al 2016	!	+	+	-	!	!
Geng et al 2022	!	+	+	-	!	-
Giraudier et al 2020	!	+	+	-	+	-
Gurtubay et al 2021	+	+	+	+	+	+
Hasan et al 2015	+	!	+	+	+	!
Hein et al 2013	!	+	+	-	!	-
Huang et al 2014	+	+	+	+	+	+
Jacobs et al 2015	+	+	+	+	+	+
Jiao et al 2020	+	+	!	+	!	!
Jonkees et al 2018	!	-	-	-	!	-
Kaut et al 2019	!	-	-	-	!	-
Konjusha et al 2022	!	-	-	-	!	-
Kutlu et al 2020	-	-	-	-	!	-
Li et al 2022	+	+	+	+	+	+
Machetanz et al 2021	!	!	+	!	+	!
Manning et al 2019	!	-	+	!	+	-
Maraver et al 2020	+	!	+	-	!	!
Pihlaja et al 2020	!	-	-	-	!	-
Ricci et al 2020	!	-	-	+	+	-
Rong et al 2014	+	+	+	+	+	+
Rong et al 2015	+	+	+	+	!	!
Sclocco et al 2019	+	!	+	+	+	!
Sclocco et al 2020	+	+	+	+	+	+
Sellaro et al 2015	!	-	-	-	!	-
Sellaro et al 2017	+	-	+	+	+	-
Sharon et al 2020	!	-	+	-	!	-
Steenbergen et al 2015	+	!	+	+	+	+
Straube et al 2015	+	+	+	+	+	+
Sttavrakis et al 2022	+	+	+	+	+	+
Sun et al 2021	+	!	+	!	+	!
Takkar et al 2020	!	+	+	-	!	-
Tran et al 2019	+	+	+	+	+	+
Ventura-Bort et al 2018	!	+	+	-	+	-
Warren et al 2019	-	-	-	-	!	-
Wu et al 2020	+	+	+	+	+	+
Zhu et al 2021	+	+	+	+	+	+

+ Low risk
! Some concerns
- High risk

D1 Randomisation process
D2 Deviations from the intended interventions
D3 Missing outcome data
D4 Measurement of the outcome
D5 Selection of the reported result
