# Supplementary material for: Safety of transcutaneous auricular vagus nerve stimulation (taVNS): a systematic review and meta-analysis
Source: Sci Rep. 2022 Dec 21;12:22055. doi: 10.1038/s41598-022-25864-1 (PMC9772204; doi:10.1038/s41598-022-25864-1)
Supplement: Supplementary file 6 — Supplementary Information 6. [file 41598_2022_25864_MOESM6_ESM.pdf]

Safety of transcutaneous auricular vagus nerve stimulation(taVNS): A systematic review and meta-analysis

Angela Yun Kim1#, Anna Marduy2,3#, Paulo S. de Melo#3,4, Anna Carolyna Gianlorenco3,5, Chi Kyun g Kim6, Hyuk Choi7,8, Jae-Jun Song1,8, and Felipe Fregni3

#equally contributed authors

SUPPLEMENTARY MATERIAL 5

Evidence-based taVNS adverse events questionnaire

Evidence-based taVNS adverse events questionnaire

Session ________________

**Do you experience any of the following sym ptoms or side effects?**	**Enter a value in the space below (1, absent; 2, mild; 3, moderate; 4, severe) guided by the CTCAE**	**If present: Is this related to taVNS? (1, none; 2, remote; 3, possible; 4, probable; 5, definite)**	**Have you had this before? When has it started? For how long has it been present?**
**Ear pain**			
**Headache**			
**Tingling**			
**Dizziness**			
**Skin redness**			
**Fatigue**			
**Prickling**			
**Pressure**			
**Itching**			
**Unpleasant feeling**			
**Others (specify) ______________**			
